# Endogenous ether lipids differentially promote tumor aggressiveness by regulating the SK3 channel

**DOI:** 10.1016/j.jlr.2024.100544

**Published:** 2024-04-18

**Authors:** Marion Papin, Delphine Fontaine, Caroline Goupille, Sandy Figiel, Isabelle Domingo, Michelle Pinault, Cyrille Guimaraes, Nina Guyon, Pierre François Cartron, Patrick Emond, Antoine Lefevre, Maxime Gueguinou, David Crottès, Paul-Alain Jaffrès, Lobna Ouldamer, Karine Maheo, Gaëlle Fromont, Marie Potier-Cartereau, Philippe Bougnoux, Aurélie Chantôme, Christophe Vandier

**Affiliations:** 1 Niche, Nutrition, Cancer & Oxidative metabolism (N2COx), UMR 1069, INSERM, University of Tours, Tours, France; 2 Department of Gynecology, CHRU Bretonneau, Tours, France; 3 CRCINA-INSERM 1232, Equipe « Apoptose et Progression tumorale », Nantes, France; 4 iBrain, UMR 1253, INSERM, Université de Tours, Tours, France; 5 Nuclear medicine in vitro department, CHRU Bretonneau, Tours, France; 6 Laboratoire Chimie Electrochimie Moléculaires et Chimie Analytique (CEMCA), UMR 6521, CNRS, University of Brest, Brest, France; 7 Department of Pathology, CHRU Bretonneau, Tours, France

**Keywords:** ether lipids, miRNA, potassium channels, SK3 channel

## Abstract

SK3 channels are potassium channels found to promote tumor aggressiveness. We have previously demonstrated that SK3 is regulated by synthetic ether lipids, but the role of endogenous ether lipids is unknown. Here, we have studied the role of endogenous alkyl- and alkenyl-ether lipids on SK3 channels and on the biology of cancer cells. Experiments revealed that the suppression of alkylglycerone phosphate synthase or plasmanylethanolamine desaturase 1, which are key enzymes for alkyl- and alkenyl-ether-lipid synthesis, respectively, decreased SK3 expression by increasing micro RNA (miR)-499 and miR-208 expression, leading to a decrease in SK3-dependent calcium entry, cell migration, and matrix metalloproteinase 9–dependent cell adhesion and invasion. We identified several ether lipids that promoted SK3 expression and found a differential role of alkyl- and alkenyl-ether lipids on SK3 activity. The expressions of alkylglycerone phosphate synthase, SK3, and miR were associated in clinical samples emphasizing the clinical consistency of our observations. To our knowledge, this is the first report showing that ether lipids differentially control tumor aggressiveness by regulating an ion channel. This insight provides new possibilities for therapeutic interventions, offering clinicians an opportunity to manipulate ion channel dysfunction by adjusting the composition of ether lipids.

SK3 channels, members of the small conductance calcium-activated potassium channels family, have been identified as promoters of tumor aggressiveness (1). SK3, along with SK1 and SK2 channels, are expressed in specific cells types (e.g., brain, smooth muscles, and, as recently shown, heart cells) and participate in cell excitability by controlling muscle contraction and cellular secretion. When SK3 is expressed in cancer cells, it strongly increases their capacity to migrate and invade a matrix similar to the physiological extracellular matrix (1). Thus, cancer cells appear to hijack the physiological functions of SK3 found in excitable cells to promote their ability to migrate. By using mouse models of metastatic breast cancers (including orthotopic xenografts), we have demonstrated that SK3 channel promotes the development of metastases (1). Our previous study also identified a new molecule belonging to the class of synthetic alkyl ether lipids (ELs), 1-O-hexadecyl-2-O-methyl-sn-glycero-3-lactose (ohmline), which inhibits SK3 channel activity and abolishes the development of metastases (1, 2). This prompted us to investigate the role of endogenous EL in the regulation of SK3. Endogenous ELs exist as triglycerides or phospholipids and contain a long fatty alcohol chain linked to the *sn*-1 position of the glycerol unit by an ether bond. Within cell membranes, they form alkyl (with a saturated ether bond) or alkenyl phospholipids (also referred to as plasmalogens, with a vinyl-ether bond). The distribution of EL varies across tissues, with alkenyl-EL generally more abundant than alkyl-EL. Both EL species are enriched in the heart, brain, and, to a lesser extent in muscle, and adipose tissues (3, 4). Interestingly, they are generally found in greater amounts in tumors, including breast tumors, than in normal tissues (5). However, the physiological roles of these ELs have not been elucidated so far. Moreover, it remains unknown whether alkyl and alkenyl-EL exert distinct biological effects (6), which prompted us in this work to determine whether alkyl- and alkenyl-EL had the same role in the regulation of SK3.

One study showed that alkylglycerone phosphate synthase (AGPS), the key enzyme in natural EL synthesis, was overexpressed in breast cancer tissues compared to noncancerous tissues (5, 6). AGPS is a peroxisome enzyme catalyzing the conversion of acyl-glycerone-3-phosphate to alkyl-glycerone-3-phosphate, replacing the fatty acid in the *sn*-1 position of the glycerol unit with a fatty alcohol, thus forming the first precursor for EL. Several cellular models have already shown that AGPS knockdown using siRNA decreases the amount of cellular natural EL by 60–80% and results in a decrease in cell migration (7, 8). AGPS therefore seems to be a prime target for studying the regulation of SK3 channel by endogenous EL (alkyl-EL and alkenyl-EL). More recently, the gene *TMEM189* coding for plasmanylethanolamine desaturase 1 (PEDS1), the key enzyme for alkenyl-EL synthesis, was identified. This gives us the opportunity to distinguish between alkyl-EL and alkenyl-EL, whose discrimination has so far been a major challenge (9, 10).

In this report, we took advantage of these two key enzymes producing EL to study the effects of alkyl and alkenyl-EL on the SK3 channel expression and activity and its associated biological activities.

Here, we showed that the suppression of AGPS and PEDS1 decreased SK3 expression through micro RNA (miR)-499 and miR-208, leading to a decrease in SK3-dependent constitutive calcium entry (CCE), cell migration, and matrix metalloproteinase 9 (MMP9)-dependent cell adhesion and invasion. We then identified alkyl- and alkenyl-EL that promoted SK3 expression and found a differential role of alkyl- and alkenyl-EL on SK3 activity. The expressions of AGPS, SK3, and miR were found to be correlated in clinical samples, which emphasize the clinical consistency of our observations.

## Materials and Methods

### Patients and samples

All human studies abide by the Declaration of Helsinki principles.

#### Cohort 1

The study cohort consisted of 50 female patients presenting with invasive breast cancer, scheduled for breast surgery at the University Hospital of Tours in 2012. Samples were stored in liquid nitrogen to avoid degradation and conserved in the tumor collection (Declared to French ministry of Health with n° DC2008-308), and the study was performed with approval of the Tours University Review Board. Histological slides and reports of all patients were reviewed by an expert pathologist. All less frequent phenotypes (triple negative and HER2+++ tumors) were selected, and patients with positive hormone-receptor tumor phenotypes (luminal A and B) were matched according to the age distribution of patients with the less frequent phenotypes. The characteristics of patients and tumors are reported in Supplemental Table S1.

#### Cohort 2

The study was performed on tumor samples from 78 women treated for invasive breast carcinoma by surgery and chemotherapy between 2012 and 2014 at the University Hospital of Tours (Tours, France). The study was approved by the Ethics committee of the hospital, was conducted in accordance with recognized ethical guidelines, and written informed consent was obtained from all patients. The characteristics of patients and tumors are reported in Supplemental Table S2.

#### Cohort 3

Samples were collected from patients treated at the “Institut de Cancérologie de l’Ouest” (ICO, http://www.ico-cancer.fr). All patients recruited gave signed informed consent. All the samples collected and the associated clinical information were registered in the database (N° DC-2018-3321) and validated by the French research ministry. Biological resources were stored at the “Centre de Ressources Biologiques-Tumorothèque” (ICO, Saint-Herblain, France) (11). All clinical and radiologic data were collected from the electronic medical records stored at the ICO. The characteristics of patients and tumors are reported in Supplemental Table S3.

### Cell lines and reagents

MDA-MB-435s (HTB-129), PC3 (CRL-1435), and C4-2 (CRL-3314) cancer cell lines were purchased at the American Type Culture Collection (LGC Standards, Molsheim, France). A673 (CRL-1598) cells were a kind gift from Franck Verrechia (INSERM UMR1307, France) and cultured in DMEM (Lonza, Basel, Switzerland), supplemented with 10% FBS (Gibco). PC3 and C4-2 cells were cultured in RPMI (Gibco) supplemented with 10% FBS, whereas MDA-MB-435s cells were cultured in alpha Modified Eagle’s Medium (Gibco) supplemented with 5% FBS. MDA-MB-435s cell line has been transduced by a lentivector, which contains a scrambled shRNA sequence (SK3^+^ cell) or a shRNA sequence targeting SK3 expression (SK3^-^ cell). These cells have been characterized in a previous study (12). HEK-hSK3 cells characterized previously in patch-clamp experiment (13) are cultured in DMEM supplemented with 10% fetal bovine serum. Mycoplasma contamination was regularly evaluated, and thanks to MycoAlert Mycoplasma detection kit (Lonza, Levallois-Perret, France).

Apamin was purchased from Sigma-Aldrich (Bristol) and dissolved in water. Ohmline was synthesized as described previously (12) and dissolved in 60% DMSO/40% ethanol (EtOH) (v/v). Details on the lipids tested are in Supplemental Table S4.

### Immunohistochemistry on tissue microarray

#### TMA construction

Tissue microarrays (TMAs) were constructed using formalin-fixed paraffin-embedded tissue samples. For each case, 3 cores 0.6 diameter were transferred from the selected areas to the recipient block, using a TMA workstation (Manual Tissue Arrayer MTA Booster, Alphelys, France). Serial 3 μm sections of the TMA blocks were used for immunohistochemistry. One section on 10 was stained with hematoxylin-Manual Tissue Arrayer MTA eosin to check that the cores adequately represented diagnostic areas.

#### Immunohistochemistry

Slides were deparaffinized, rehydrated, and heated in citrate buffer pH 6 for antigenic retrieval. After blocking for endogenous peroxidase with 3% hydrogen peroxide, the primary antibodies were incubated as described in Supplemental Table S5.

Immunohistochemistry was performed using the streptavidin-biotin-peroxidase method with diaminobenzidine as the chromogen (Kit LSAB, Dakocytomation, Glostrup, Denmark). Slides were finally counterstained with hematoxylin. Negative controls were obtained after omission of the primary antibody or incubation with an irrelevant antibody. Staining was scored as negative or positive.

### Western blot

Whole-cell lysates were prepared with 2% sodium dodecyl sulphate and protease inhibitor cocktail (P2714, Sigma-Aldrich, France). Protein concentration was determined using BCA protein assay kit (Thermo Fisher Scientific). Proteins (25 μg) were separated by denaturing SDS-PAGE (4–15% Mini-PROTEAN® TGX Stain-Free™ Protein Gels, Bio-Rad) and transferred onto polyvinylidene fluoride membranes (Trans-Blot Turbo Mini Transfer Packs, Bio-Rad). Dilution (in 1% nonfat dry milk, 1X TBS 0.1% Tween-20), incubation conditions, and reference of primary antibodies used are described in Supplemental Table S5. Antibody binding was revealed with an anti-rabbit (1:10,000; Jackson Immuno-Research Laboratories) IgG coupled to horseradish peroxidase, using ECL chemiluminescence kit (Clarity Western ECL substrate, Bio-Rad) and read on ChemiDoc Imaging System (Bio-Rad). Band quantification was performed on Image Lab software (Bio-Rad) using total protein stain as normalization.

### siRNA, miRNA, and plasmid transfection

Cells were seeded in 6-wells plates at 200,000 cells and transfected 24 h later with 40 nM of siRNA by using lipofectamine RNAimax (Invitrogen, France) or with 10 nM of LNA™ miRNA mimics (hsa-miR-499a-5p, hsa-miR-208a-3p, negative control miRCURY LNA, Qiagen, Courtaboeuf, France) by using HiPerfect transfection reagent (Qiagen), following manufacturer’s instructions. siRNA sequences are detailed in Supplemental Table S6 and were synthetized by Eurogentec (Belgium). The construct of human SK3 (pPRIpuhSK3) was kindly provided by Dr P. Martin (CNRS, UMR 6543, Nice, France). The pore motif, GYG, was altered to AAA by site-directed mutagenesis leading to a nonconductive or nonpore SK3 (pPRIpuhSK3np). This construct was produced and sequenced by ProteoGenix (France). Cells were seeded in 6-wells plates at 350,000 cells and transfected 24 h later with 0.25 μg of pPRIpu (CTL), pPRIpuhSK3 (SK3WT), or pPRIpuhSK3np (SK3np) by using TransIT-2020 reagent (Mirus Bio LLC), following manufacturer’s instructions.

### RT-qPCR

RNA from cells was extracted with the NucleoSpin RNA kit (Macherey-Nagel, Hoerdt, France). RNA from tumors was extracted with Maxwell RSC simplyRNA Tissue Kit (Promega). Reverse transcriptions of 500 ng of RNA were performed with PrimeScript RT-PCR kit (Takara, Ozyme, France). Quantitiative polymerase chain reaction (qPCR) were performed on the CFX Connect Real Time System (Bio-Rad, France) using TB Green® Premix Ex Taq™ (Takara, France), following manufacturer’s instructions. Quantity of mRNA was analyzed using the ΔΔCT method normalized to the housekeeping genes HPRT and ALAS-1 for cell experiments and HPRT and TBP for tissue experiments. Primers were purchased at Eurogentec (Seraing, Belgium), and their sequences are detailed in Supplemental Table S7. The miRNA were extracted with miRNEasy mini kit (Qiagen, Courtaboeuf, France) and were reverse transcribed using the miRScript II RT kit (Qiagen) and analyzed by qPCR with the miScript SYBR Green PCR Kit (Qiagen) using the specific hsa-miR miScript Primer Assays (Qiagen) according to the manufacturer's instructions. miRs expression fold changes were calculated using the 2-ΔΔCt formula and SNORD61 and RNU6-2 as normalizers according to the manufacturer’s instructions.

### Reporter gene constructs and luciferase assay

*KCNN3* promoter sequence has been delineated following publication of Sun *et al.* that described limits of functional promoter (2,540 bp) (Sun, G *et al.*, J. Hum. Genet. 2001). Nhe1 and Nde1 sites have been created for actual and further cloning purposes. For convenient cloning, a DNA synthetic fragment comprising promoter from Nhe1 site to internal luciferase Mre1 site has been generated and inserted in Nhe1 and Mre1 sites of pGL4.17 cloning reporter vector (Promega). Stable transfection was performed with TransIT-2020 reagent (Mirus Bio LLC) and G418™ selective antibiotic (Sigma-Aldrich). The MDA-MB-435s-*KCNN3* promoter-luc cells were transfected with siAGPS for 48 h and 72 h. Cells were seeded in a 24-wells plate at 40.000 cells 24 h before measurements. Cells were lysed with passive lysis buffer (Promega), and reporter assays were measured with luciferase assay system (E1500, Promega) according to the manufacturer's instructions. The quantification of proteins contained in samples was assessed and bioluminescence was normalized to protein quantity.

### Cross-linking immunoprecipitation

Cross-linking immunoprecipitation experiments were performed using a RiboCluster Profiler RIP-Assay kit (CliniScience, Nanterre, France) according to the manufacturer's protocol plus some modifications.

Cross-linking step was performed on ice by cells irradiation with UV light (365 nm) at 150 mJ/cm2, and cytoplasmic lysates were obtained according to the manufacturers.

Prestep of preparation of antibody-immobilized Protein/A/G agarose beads was performed by incubating 15 μg of control antibody (IgG, Abcam, Amsterdam, Netherlands) or GW182 antibody (CliniSciences, Nanterre, France).

Cellular lysate and antibody-immobilized Protein/A/G agarose beads were incubated overnight at 4°C with rotation. Separation method was next used to isolate large and small RNAs according to the manufacturer’s instructions. Reverse transcription quantitiative polymerase chain reaction were next performed to identify endogenous mRNAs and miRNAs coimmunoprecipitated with GW182. Relative levels of enrichment were calculated using Ct values (immunoprecipitation anti-IgG, immunoprecipitation anti-GW182 and input) and the 2ΔΔCt formula.

For cell viability assay, see Supplemental Text S1.

### Cell migration and invasion assays

Cell migration and invasion were assessed by using 8 μm pore size polyethylene terephthalate membrane cell culture inserts without (Falcon, #353097) or with Matrigel™ (Corning, #354480). After 48 h of transfection, cells were seeded at 40,000 (migration) or 50,000 (invasion) cells per insert. Migration was performed for 24 h and cells were fixed with cold methanol. Cells that did not cross the membrane were removed with a cotton ball, and nuclei were stained with DAPI (Sigma-Aldrich). Nine pictures per insert were taken and nuclei were automatically counted (14).

### Cell adhesion assays

Cell adhesion by tapping was assessed by seeding 40,000 cell per well on a 96-wells plate coated with 6 μg/cm^2^ of fibronectin (Sigma-Aldrich, #F1141). After 2 h, culture medium was removed and the plate was firmly tapped once on the bench surface covered with absorbent paper and PBS was added to the wells. This procedure was repeated three times. The remaining cells were fixed with EtOH and their nuclei were stained. Nine pictures per insert were taken and the nuclei were automatically counted (14). To assess an increase in adhesion, impedance measurements were performed with the xCELLigence real-time cell analysis DP (Agilent technologies) system after seeding 20,000 cells per well in 16-wells E-plate (Agilent technologies, France) coated with 6 μg/cm^2^ of fibronectin (Sigma-Aldrich, #F1141). The cell index, corresponding to the impedance at any time point minus the impedance without cells was monitored every 4 min for 150 min.

### Calcium entries measurements

For CCE, cells were seeded in 6-wells plates at 200,000 cells per well and 24 h after cells were incubated with the ratiometric calcium probe Fura-2 AM (F1201 – 1 mg, Molecular Probes) at 1 μM for 45 min at 37°C. Then medium was removed, cells detached with EDTA, and suspended in OptiMEM (Life Technologies, Saint Aubin, France). After centrifugation, OptiMEM was removed and cells were resuspended into 2 ml of free-calcium physiological saline solution (NaCl 140 mM, KCl 4 mM, MgCl_2_ 2 mM, Hepes 10 mM, glucose 11.1 mM, and EGTA 1 mM, pH: 7.4) in a magnetically stirred cuvette and after 10 s of measurements 2 mM of calcium were added. Cytosolic calcium variations were obtained by a spectrofluorometer (F-2710 FL, Hitachi/VWR, Fontenay-sous-Bois, France) by measuring fluorescence at 510 nm after excitation of Fura-2 at 340 and 380 nm. The 340/380 nm ratio is proportional to cytosolic calcium concentration. For more details, see previous study (1).

For store-operated calcium entry (SOCE), see Supplemental Text S1.

### Patch clamp experiments

Experiments were performed using the conventional whole-cell recording configuration of the patch-clamp technique with an extracellular solution and an intracellular pipette solution previously described (Girault *et al.* 2011). Stable transfection of pPRIpuhSK3 was performed in HEK293T cells by using TransIT-2020 reagent (Mirus Bio LLC) as transfection reagent and puromycin as selective antibiotic (Sigma-Aldrich) as described previously (13). Whole-cell currents in HEK293T-hSK3 cells were generated by ramp protocol from +100 to −100 mV in 500 ms (4 s intervals) from a constant holding of 0 mV with a pCa 6. Whole-cell recordings of membrane currents were made under vehicle application during 4 min then with an EL application.

### Bioinformatics analyses

RNA-Seq data from prostate adenocarcinoma and normal tissue were generated by The Cancer Genome Atlas Research Network (http://cancergenome.nih.gov/) and Genotype Tissue Expression consortium, respectively (15). The Genotype-Tissue Expression Project was supported by the Common Fund of the Office of the Director of the National Institutes of Health, and by NCI, NHGRI, NHLBI, NIDA, NIMH, and NINDS. Logarithmic transformed normalized data for *KCNN3* and AGPS (expressed as transcript per million units) from TCGA-PRAD and Genotype-Tissue Expression dataset were obtained from UCSC (16) Xena Browser (https://xena.ucsc.edu).

### EL liposome preparation and supplementation

Liposomes were prepared according to a method adapted from (17). Briefly, 1 mg of EL was diluted in 1 ml of chloroform in a glass tube. Chloroform was evaporated until complete dryness. One milliliter of sterile water was added to the tube, which was closed and let at 4°C overnight to let the lipid film hydrate. The resulting solution was sonicated three times at 40°C for 10 min and vortexed. Liposomes were considered usable when no visible aggregate remained and stored at 4°C.

To determine the relevant dose for EL supplementation, the amount of EL in FBS was first measured by high-performance TLC (HPTLC) according to the method described previously (18), slightly modified from the method described hereafter to quantify separately alkyl- and alkenyl-EL. We estimated that FBS contained on average 1.9 μg/ml of alkyl-EL and only traces of alkenyl-EL (N = 3). Cells were treated with water (vehicle control) or liposomes at 20 μM in culture media daily for up to 96 h, representing a dose of EL roughly 500 times greater than the dose provided by FBS. The liposomes were sonicated for 5 min at 40°c and vortexed for 5 s right before treatment.

### EL quantification (HPTLC) and composition (mass spectrometry)

#### High-performance TLC

Cells were extracted according to the Bligh and Dyer method (19). Samples were solubilized in an ether/hexane (80/20, v/v) mix and nonether links were reduced by Vitride® (sodium hydride and aluminium) (0.5 ml Red-Al® for 10 mg total lipids) according to a described method (20). Reaction is neutralized by a mix EtOH/water (20/80, v/v) in ice. Obtained products from the ether phase were dried and r-solubilized in chloroform/methanol (2/1, v/v) at the concentration of 20 mg/ml. The silica gel plate for HPTLC premigrated in chloroform/methanol (1/1, v/v), samples were separated by migration in chloroform/acetone (90/10, v/v) for 30 min and were revealed in a bath of sulfuric acid/EtOH (10/90, v/v) for 1 min. Plate was heated at 140°C for 11.5 min to carbonize organic substances. A range of batyl (1-O-octadecyl-*sn*-glycerol) from 1 to 10 μg migrated with samples. Bands were visualized by the TLC visualizer (Camag) and quantified using the batyl range. Quantities of EL were expressed in microgram per million of cells and normalized to control condition.

#### Mass spectrometry

The dry residue was reconstituted with 100 μl of a 6:3:1 mix of acetonitrile/water/isopropanol, followed by centrifugation (15,000 *g*, 10 min, 4^°^C) before mass spectrometry analysis. Liquid chromatography-high resolution mass spectrometry analysis was realized following already published method (21). For more detail, see Supplemental Text S1.

Data processing: Briefly, features were automatically detected using eXtensible Computational Mass Spectrometry online. Manual verification of the features detected was performed. In every sample, the intensity of each feature was normalized to the sum of all features’ intensities in the sample and expressed as the normalized peak area. An algorithm was used to identify matching isotopes and only M+0 isotopes were kept. When possible, lipids were annotated according to their retention time and *m/z* using an in-house database builds with MS/MS data. EL identified are putatively annotated as follows: “PC” or “PE” stands for the phosphatidylcholine (PC) or phosphatidylethanolamine (PE) polar heard, respectively. “O-” means ether bond (alkyl-EL) and “P-”, vinyl-ether bond (alkenyl-EL). When possible, both *sn*-1 and *sn*-2 chains are annotated with their carbon chain length, followed by the number of unsaturations, with the first chain being the ether-linked one, followed by the *sn*-2 fatty acid. Some species are annotated with the sum of both their *sn*-1 and *sn*-2 chains. As a general rule due to their abundance described in the literature, EL with PC polar head are putatively identified as alkyl-EL and EL with PE polar head with at least one unsaturation as alkenyl-EL. Noteworthily, as an example, a PC(O-16:1/20:4) could be a PC(P-16:0/20:4) and a PE(P-18:0/22:6) could be a PE(O-18:1/22:6).

### Statistics

Data were represented as median ± interquartile range, with N the number of experiments and n the number of replicates. For experiments with two conditions, the Mann–Whitney test was used and for those with more than two conditions, the Kruskal–Wallis test with Dunn’s correction was used. When working with paired samples, Wilcoxon matched-pairs signed rank test was used. Differences were considered significant when *P* value <0.05 (the software used for statistical analysis was GraphPad Prism). For immunohistochemistry assay on TSA, statistical analyses were carried out with StatView, version 5.0, software (Abacus Concepts, Berkeley, CA). Comparison between groups was performed using the χ2 test or the Fisher exact test. For heatmap representation, the normalized peak area of each EL was centered and scaled across all the samples, converting them to a Z-score and plotted as a heatmap using R (22, 23).

## Results

### Coexpressions of AGPS and SK3 in breast biopsies

**Fig. 1:**
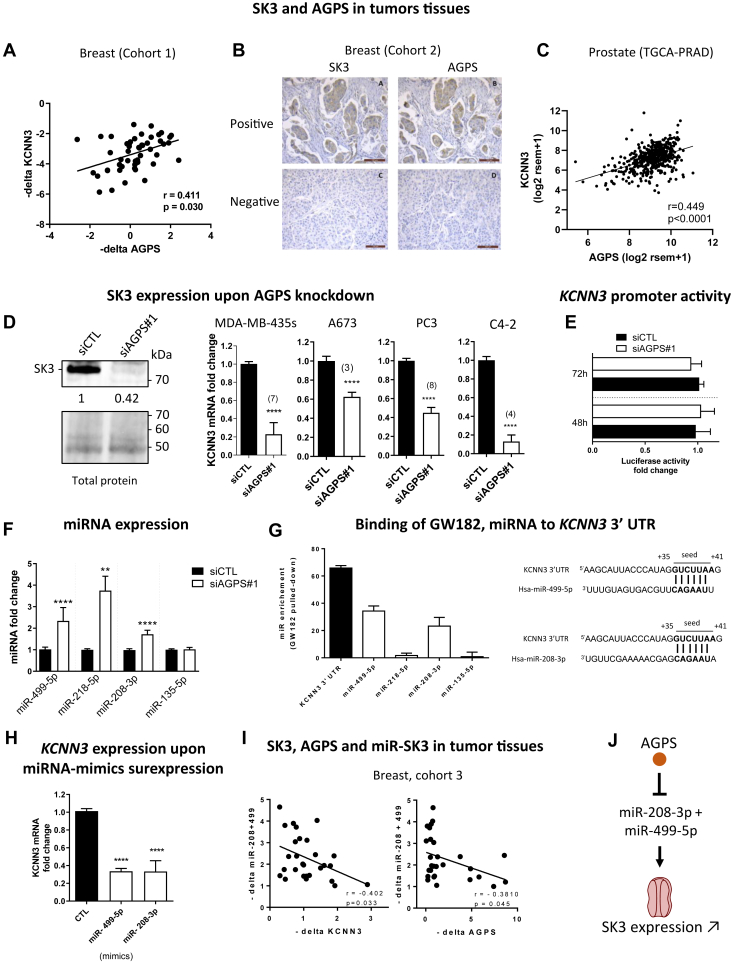
AGPS promotes SK3 expression by regulating miRNA: evidence in human cancer cells and tissues. A–C: AGPS correlates with SK3 in invasive breast carcinoma and prostate adenocarcinoma. A: Scatter plot representing the Pearson’s correlation analysis between *AGPS* and *KCNN3* mRNA (translating SK3 protein) quantified by RT-qPCR. Cohort 1, N = 50, r = 0.4, ∗*P* < 0.05. B: Representative figures of immunohistochemical staining for AGPS and SK3 in human breast cancer tissues (scale bar = 100 μm). Top: most tumors positive for SK3 were also positive for AGPS (23 on 28). Bottom: the majority of SK3 negative cases were also negative for AGPS (30 on 50). Cohort 2, N = 78, Chi2, ∗∗∗∗*P* = 0.0002. No correlation between AGPS staining and histoprognostic grade SBR or with the lymph node status was found (*P* = 0.36). C: Scatter plot representing the Pearson’s correlation of the mRNA expression of *KCNN3* in function of the mRNA expression of AGPS in prostate adenocarcinoma. TGCA-PRAD dataset, N = 495, r = 0.4, ∗∗∗∗*P* < 0.0001. D: AGPS is critical for SK3 expression in cancer cells. Left: SK3 protein level studied in stain-free Western-blot is highly decreased 72 h after transfection with siAGPS#1 compared to siCTL in MDA-MB-435s cells. SK3 detection band area was normalized to the total protein signal of the lane and then relativized to the siCTL condition (N = 4). Right: *KCNN3* mRNA level analyzed by RT-qPCR is decreased 72 h after transfection with siAGPS#1 compared to siCTL in MDA-MB-435s, A673, PC3, and C4-2 cells (median ± interquartile range, Mann–Whitney test, ∗∗∗∗*P* < 0.0001). The numbers in brackets indicate the number of independent experiments. E: AGPS knockdown does not affect *KCNN3* promoter activity. *KCNN3* promoter activity studied by reporter luciferase assay was performed 48 h (N = 4) and 72 h (N = 6) after siAGPS#1 transfection in MDA-MB-435s cells. Data are relativized to siCTL condition (median ± interquartile range, N = 6, Mann–Whitney test, not significant). F: AGPS knockdown increases the expression of putative miRNA–targeting *KCNN3* mRNA. miR-499-5p (N = 7), miR-208-3p (N = 4), miR-218-3p (N = 3), and miR-135-5p (N = 3) were quantified by RT-qPCR 48 h after transfection with siAGPS#1 in MDA-MB-435s cells. Data are relativized to siCTL condition (median ± interquartile range, Mann–Whitney test, ∗∗∗∗*P* < 0.0001, ∗∗*P* < 0.01). G: miR-208-3p and miR-499-5p bind to 3′ UTR *KCNN3* mRNA. Left: organization of endogenous *KCNN3* mRNA, miRNAs, and GW482 (RNA-binding protein) within RNA-induced silencing complex (RISC) assessed by cross-linking immunoprecipitation in MDA-MB-435s cells. The binding of GW182, miR-499a-5p, or miR-208-3p to the 3′ UTR *KCNN3* mRNA were detected. In opposite, the binding were not detected with miR-218-3p and miR-135-5p (N = 3, median ± interquartile range). Right: alignment of the sequences of miR-499-5p and miR-208-3p with their target site in the 3′ UTR of *KCNN3* mRNA. The binding site is the same for both miRNAs and is highly conserved among mammals. H: miR-208-3p and miR-499-5p mimics downregulate *KCNN3* mRNA. KCNN3 mRNA was quantified by RT-qPCR 48 h after transfection with miR-499-5p or miR-208-3p mimics and relativized to the mimic control condition in MDA-MB-435s cells. (N = 4, median ± interquartile range, Kruskal–Wallis test, *P* < 0.0001 and post hoc Dunn’s test (compared to control condition),∗∗∗∗*P* < 0.0001). I: AGPS and *KCNN3* mRNA are inversely correlated with miR-208-3p and miR-499-5p in breast carcinoma. *AGPS*, *KCNN3*, miR-499-5p, and miR-208-3p were quantified by RT-qPCR. Graphics show Pearson’s correlation analysis. Cohort 3, N = 28, *KCNN3* versus miR-208-3p+miR-499-5p: r = −0.40, ∗*P* < 0.05, AGPS versus miR-208-3p and miR-499-5p: r = 0.38, ∗*P* < 0.05. J: Schematic representation of SK3 regulation by AGPS. AGPS, alkylglycerone phosphate synthase; miRNA, micro RNA; qPCR, quantitiative polymerase chain reaction; RT-qPCR, reverse transcription quantitiative polymerase chain reaction; SBR, Scarff Bloom et Richardson; TCGA, The Cancer Genome Atlas; UTR, untranslated region.

To study the expression of AGPS and SK3 in breast cancers, we analyzed *AGPS* and *KCNN3* mRNA (coding for SK3 protein) expressions in 50 invasive breast cancer biopsies (cohort 1). Figure 1A shows a significant correlation between *AGPS* and *KCNN3* mRNA. To confirm this observation, immunohistochemical staining against AGPS and SK3 proteins was carried out on a TSA (serial 3 μm sections) of 78 invasive breast carcinoma specimens (cohort 2). As illustrated in Fig. 1B, we observed that the AGPS protein was expressed in 43 tumors out of 78, the SK3 protein was positively stained in 38 cases, and a significant correlation between AGPS and SK3 protein expression was found. Moreover, according to the gene expression analysis of the Cancer Genome Atlas (https://portal.gdc.cancer.gov/) database, a similar correlation between *AGPS* and *KCNN3* mRNA was observed in prostate tumors, extending these observations beyond breast cancer (Fig. 1C).

### AGPS promotes SK3 by regulating miR-499 and miR-208

Since the key function of AGPS is to synthesize EL and that the SK3 channel was previously found to be regulated by EL analogs (2), we then investigated whether knocking down the AGPS affected SK3 expression in various cancer cell lines (breast and prostate cancer, osteosarcoma). First, we checked the efficiency of siRNA and found that siAGPS#1 inhibited more than 90% the *AGPS* mRNA and 73% of the protein 72 h after transfection (Supplemental Fig. S1, A–D). As expected, the HPTLC results showed that knocking down AGPS significantly decreased the quantity of EL by 81% (N = 5, Mann–Whitney test, *P* = 0.017, Supplemental Fig. S23). This was associated with a large reduction of the expression of the SK3 protein and *KCNN3* mRNA in various cancer cell lines (Fig. 1D). This effect was time-dependent (Supplemental Fig. S2A), with a large effect observed 72 h post transfection in the four different cell lines tested, namely MDA-MB-435s, A-673, PC3, and C4-2 cells (Fig. 1D). Another sequence of siRNA (siAGPS#2) yielded the same results on AGPS and SK3 expressions (Supplemental Figs. S1E and S2B). It has been reported that some epithelial-to-mesenchymal transition transcription factors were found to be regulated by AGPS (8), including Zeb1 which we found to enhance *KCNN3* promoter activity (24, 25, 26). Nevertheless, we found that AGPS knockdown had no effect on the expression of various epithelial-to-mesenchymal transition transcription factors, including Zeb1 expression (Supplemental Fig. S3). In addition, AGPS knockdown had no effect on *KCNN3* promoter activity (Fig. 1E), suggesting that AGPS could control SK3 expression through a posttranscriptional regulation mechanism, such as through miRNA expression. Interestingly, miR-499a-5p (that we named hereafter miR-499), which was previously found to control *KCNN3* expression (27), increased after siAGPS transfection (Fig. 1F). Bioinformatic analyses (www.targetscan.org/vert_71/) predicted three other miR, among which miR-218-3p and miR-208a-3p (miR-208) were found to be increased by AGPS knockdown but not miR-135-3p (Fig. 1F). To determine whether these miRNAs could bind the 3′ untranslated region (UTR) of *KCNN3*, cross-linking immunoprecipitation was performed using GW182 antibodies to immunoprecipitate miR induced silencing complexes. Reverse transcription quantitiative polymerase chain reaction were subsequently performed and only the binding of miR-499 and miR-208 to the *KCNN3* 3′UTR was detected (Fig. 1G, left). Note that miR-499 and miR-208 had similar seed sequences and targeted the same site in *KCNN3* 3′UTR (Fig. 1G, right). The regulatory role of these two miRs on the expression of *KCNN3* mRNA was confirmed through the use of miR-499 and miR-208 mimics as shown in Fig. 1H. Consistent with all these results, a significant inverse correlation between *AGPS* and miR-499/miR-208, *KCNN3* and miR-499/miR-208 was observed in invasive breast carcinoma tissues (cohort 3) (Fig. 1I). All the results demonstrate that the key enzyme responsible for EL synthesis, AGPS, promoted SK3 expression by decreasing the expression of miR-499 and 208 (schematic representation in Fig. 1J).

### AGPS promotes SK3-dependent cell migration, invasion, and adhesion

**Fig. 2:**
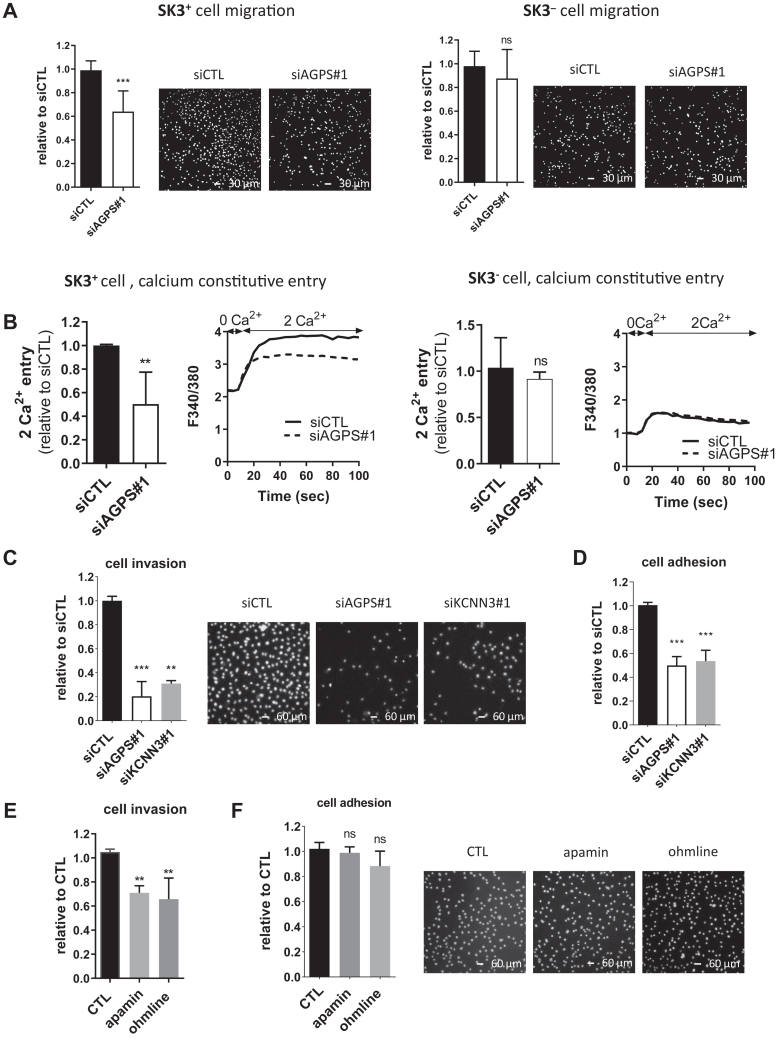
AGPS drives SK3-dependent cell migration, invasion, and adhesion. A: The ability of SK3^+^ cells to migrate is significantly reduced unlike SK3^-^ cells upon AGPS knockdown. MDA-MB-435s cells containing a sh-control (SK3^+^ cell) or a sh-SK3 (SK3^-^ cell) were transfected with siAGPS#1 and seeded in migration inserts after 48 h. Results are relative to siCTL. Representative pictures of migration inserts are shown with nuclei staining in white color. (Scale bar = 30 μm, N = 3, median ± interquartile range, Mann–Whitney test, ∗∗∗*P* < 0.001, ns = not significant). B: Constitutive calcium entry in SK3^+^ is significantly reduced unlike SK3^-^ cells upon AGPS knockdown. MDA-MB-435s cells containing a sh-control (SK3^+^ cell) or a sh-SK3 (SK3^-^ cell) were transfected with siAGPS#1 during 72 h hours before the measurement of constitutive calcium entry by using Fura-2-AM probe. Results presented as histograms are relative to siCTL. Representative recordings of fluorescence are shown. (N = 3, median ± interquartile range, Mann–Whitney test, ∗∗*P* < 0.01, ns = not significant). C and D: Cell invasion and adhesion are reduced upon AGPS or SK3 knockdown. C: MDA-MB-435s cells were transfected with siCTL (N = 4), siKCNN3#1 (N = 4), siAGPS#1 (N = 3) 2 days before cell invasion assays (24 h). Data are relativized to siCTL condition (median ± interquartile range, Kruskal–Wallis test, *P* < 0.0001 and post hoc Dunn’s test, compared to siCTL condition, ∗∗*P* < 0.01, ∗∗∗*P* < 0.001). Representative pictures of cells after cell adhesion assays. The scale bar represents 60 μm, and nuclei staining are in white color. D: MDA-MB-435s cancer cells were transfected with siCTL, siKCNN3#1, or siAGPS#1 3 days before cell adhesion assay (2 h). *P* < 0.001. Data are relativized to siCTL condition (N = 3, median ± interquartile range, Kruskal–Wallis test, *P* < 0.0001, and post hoc Dunn’s test, compared to siCTL condition, ∗∗∗*P* < 0.001. E and F: Cell invasion is slightly reduced and cell adhesion is unmodified by pharmacological inhibitors of SK3 currents. E: MDA-MB-435s cells were pretreated daily during 2 days then during invasion assay (24 h) with apamin (100 nM) or ohmline (1 μM), two inhibitors of SK3 currents. Data are relativized to control condition (median ± interquartile range, Kruskal–Wallis test, *P* < 0.0001 and post hoc Dunn’s test, compared to siCTL condition, ∗∗*P* < 0.01. F: MDA-MB-435s cells were pretreated daily during 3 days then during adhesion assay (2 h) with apamin (100 nM) or ohmline (1 μM), two inhibitors of SK3 currents. Data are relativized to control condition (N = 4, median ± interquartile range, Kruskal–Wallis test, *P* = 0.05. Representative pictures of cells after cell adhesion assays. The scale bar represents 60 μm; and nuclei staining are in white color. AGPS, alkylglycerone phosphate synthase.

In a previous study, we found that the SK3 channel has no effect on cell viability but promotes the migration of various cancer cell lines by increasing SOCE (dependent of calcium from reticulum endoplasmic) and only CCE (not dependent of intracellular calcium stores) in MDA-MB-435s cells (1, 24, 28). As expected, AGPS knockdown had almost no effect on cell viability (Supplemental Fig. S4) but decreased by 56% the migration of MDA-MB-435s, which expressed SK3 (SK3^+^ cells), and their CCE by 50% (Fig. 2A,B, left). These effects were totally abolished in MDA-MB-435s, which did not express SK3 (SK3^-^ cells) (Fig. 2A,B, right). The suppression of AGPS also had no effect on the SOCE of MDA-MB-435s (Supplemental Fig. S5A). Note that we already found that the suppression of SK3 in MDA-MB-435s reduced the capacity of the cell to migrate by 59% and decrease the CCE by 60% compared to SK3^+^ cells (1). SK3 potassium channels are associated with the Orai1 calcium channel forming an oncocomplex that promotes CCE and cell migration (29). It was observed that AGPS knockdown had no effect on Orai1 expression (Supplemental Fig. S5B) suggesting a specific control of SK3 by AGPS, leading to increased CCE and cell migration.

We subsequently investigated the role of AGPS in cell invasion and adhesion, other key cellular mechanisms promoting tumor aggressiveness. Figure 2C,D show that cancer cell invasion was reduced by 80% and cell adhesion by 50% upon AGPS knockdown. Since SK3 expression is controlled by AGPS and its role in cell invasion and adhesion remains unknown, we investigated the involvement of the SK3 channel in these cell functions. The downregulation of SK3 expression by using a siRNA directed against *KCNN3* (si*KCNN3*#1) (Supplemental Fig. S6) reduced cell invasion by 70% and cell adhesion by 46% (Fig. 2C,D), suggesting that part of AGPS-dependent cell invasion and adhesion depended on SK3 expression. Curiously, a treatment with two different SK3 channel blockers (apamin and ohmline that inhibit SK3 currents) was two times less effective in cell invasion (Fig. 2E) than si*KCNN3*#1 (Fig. 2C). This differential outcome was even more obvious when we studied the effect on cell adhesion assay since the capacity of cells to adhere remained totally unchanged with apamin or ohmline treatments (Fig. 2F). Note that we previously reported that apamin or ohmline reduced the capacity of the cell to migrate by 50% and 60%, respectively (12, 30). In order to remove any doubt on a nonspecific effect of si*KCNN3*#1, we used a second si*KCNN3* sequence (si*KCNN2*#2) that was also found to reduce cell adhesion (Supplemental Fig. S7). We then hypothesized that the differential effects of SK3 channel blockers compared to SK3 knockdown on cell adhesion could be explained by the presence of a nonpore function of SK3 that controls cell adhesion.

### A new nonpore function of SK3 promotes cell adhesion and invasion through the AGPS/SK3/MMP9 axis

**Fig. 3:**
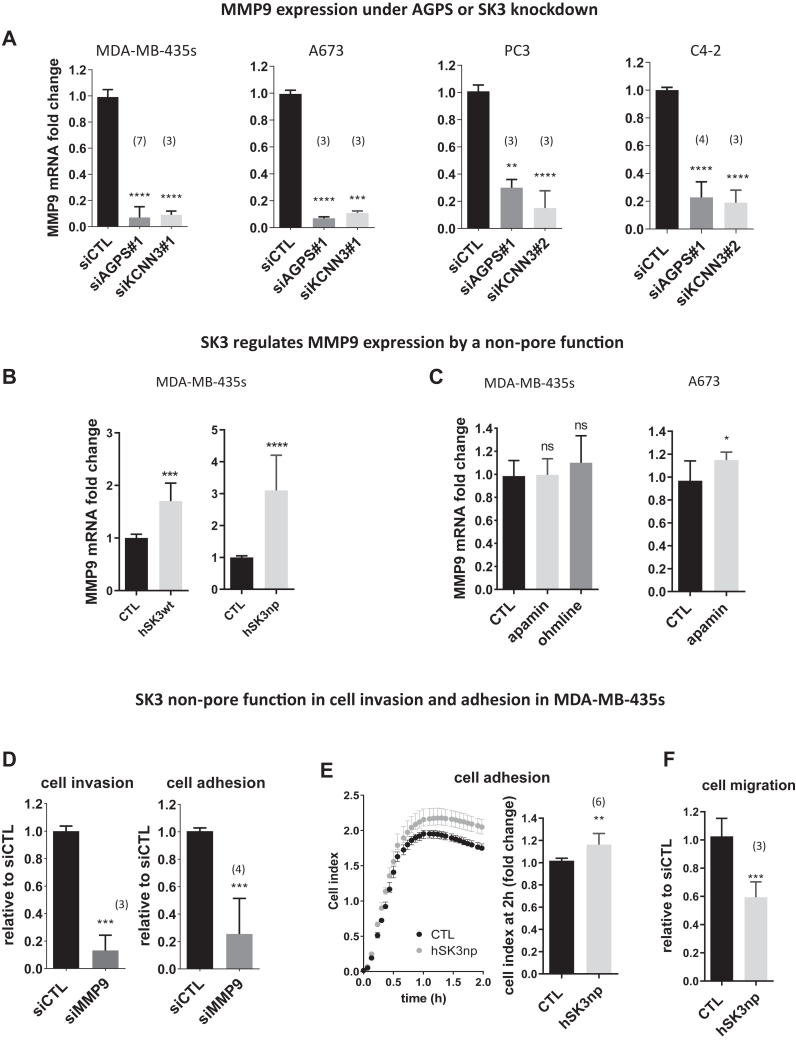
A new nonpore function for SK3 promotes cell invasion and adhesion through AGPS/SK3/MMP9 axis. A: *MMP9* expression is under the control of AGPS and SK3 expressions. *MMP9* mRNA was quantified by RT-qPCR 72 h after transfection with siCTL, siAGPS#1, siKCNN3#1, or siKCNN3#2, and relativized to siCTL condition (median ± interquartile range, Kruskal–Wallis test, *P* < 0.0001, and post hoc Dunn’s test, compared to control condition: ∗∗∗∗*P* < 0.0001, ∗∗∗*P* < 0.001, and ∗∗*P* < 0.01). The numbers in brackets indicate the number of independent experiments. B: *MMP9* expression is upregulated by the overexpression of wild-type SK3 as well as mutated pore (H425V) SK3 construct. *MMP9* mRNA was quantified by RT-qPCR 72 h after transfection of MDA-MB-435s cells with 0.25 μg control (CTL), human wild-type SK3 (hSK3wt), or human SK3np (hSK3np) plasmids. (N = 3, median ± interquartile range, Kruskal–Wallis test for the experiment with MDA-MB-435s cells, ∗∗∗*P* < 0.001,∗∗∗∗*P* < 0.0001). C: *MMP9* expression is unmodified by a treatment with SK3 inhibitors. Cells are treated daily during 3 days with apamin (100 nM) or ohmline (1 μM) and MMP9 mRNA was quantified by RT-qPCR and relativised to control condition. (N = 3, median ± interquartile range, Kruskal–Wallis test for the experiment with MDA-MB-435s cells: *P* > 0.05, Mann–Whitney test for the experiment with A-673 cells, ∗*P* < 0.05). D: Cell invasion and adhesion are under control of *MMP9* expression. MDA-MB-435s cells were transfected with siCTL or siMMP9. Invasion assay was performed for 24 h, 48 h post transfection. Adhesion assay was performed after 72h h of transfection. Data are relativized to siCTL condition (median ± interquartile range, Mann–Whitney test, ∗∗∗*P* < 0.001). The numbers in brackets indicate the number of independent experiments. E and F: Effect of mutated pore SK3 construct overexpression on cell migration and adhesion. MDA-MB-435s cells were transfected with 0.25 μg control (CTL) or human SK3np (hSK3np) plasmids. E: Adhesion assay was performed after 72 h by seeding the transfected cells in plates coated with electrodes to measure cell index every 4 min for 2 h. Left: representative graphic showing the median and the interquartile range for one experiment. Right: after 2 h, the relative cell index was significantly increased in the hSK3mut condition (median ± interquartile range, Mann–Whitney test, ∗∗*P* < 0.01). F: Cell migration assay was performed for 24 h, 48 h post transfection (median ± interquartile range, Mann–Whitney test, ∗∗∗*P* < 0.001). The numbers in brackets indicate the number of independent experiments. AGPS, alkylglycerone phosphate synthase; MMP, matrix metalloproteinase; qPCR, quantitiative polymerase chain reaction; RT-qPCR, reverse transcription quantitiative polymerase chain reaction.

Invasion-related genes such as the MMP were previously reported to be regulated by AGPS expression in cancer cells (31). If the expression of *MMP2* was decreased when AGPS was suppressed, it remained unchanged after SK3 knockdown (Supplemental Fig. S8A). In contrast, the suppression of AGPS or SK3 led to a strong reduction of *MMP9* mRNA expression in various cell lines, suggesting that AGPS controlled *MMP9* expression through the regulation of the SK3 channel (Fig. 3A). Consistent with this result, an overexpression of SK3 channel increased *MMP9* mRNA expression by 70% (Fig. 3B). In contrast, a decrease of *MMP9* expression was not observed with SK3 channel blocker treatments, while apamin treatment slightly increased *MMP9* mRNA in A673 cells (Fig. 3C). Note that this increase could be explained by an increase of SK3 expression in A673 cells observed after long-term SK3 channel blocker treatments (Supplemental Fig. S9). To explore the role of a nonpore function of SK3, we used a SK3 mutant where the pore motif, GYG, was altered to AAA leading to a nonconductive or nonpore SK3 (SK3np) (32, 33). The enforced expression of SK3np increased the expression of *MMP9* by 210% (Fig. 3B), while the suppression of *MMP9* expression reduced the cell invasion and adhesion by 80% (Fig. 3D and Supplemental Fig. S8B). In addition, the enforced expression of SK3np increased the adhesion capacity of the cells (Fig. 3E). These findings strongly suggest that SK3 promotes *MMP9* expression independently of its channel activity, resulting in a promotion of cell adhesion and cell invasion. Since part of the cell invasion capacity depends on cell migration, the effect of SK3 channel blockers observed on cell invasion (Fig. 2E) could be explained by their effect on cell migration. Indeed, we have already reported that apamin and ohmline decreased cell migration (1, 30). The reduction of cell migration induced by SK3np (Fig. 3F) supports this hypothesis since it was observed that when associated as a tetramer with endogenous SK3 protein, this construct acted as dominant negative of SK3 currents (32, 33).

### PEDS1, the key enzyme of alkenyl-EL synthesis, is involved in the AGPS/SK3/MMP9 axis

**Fig. 4:**
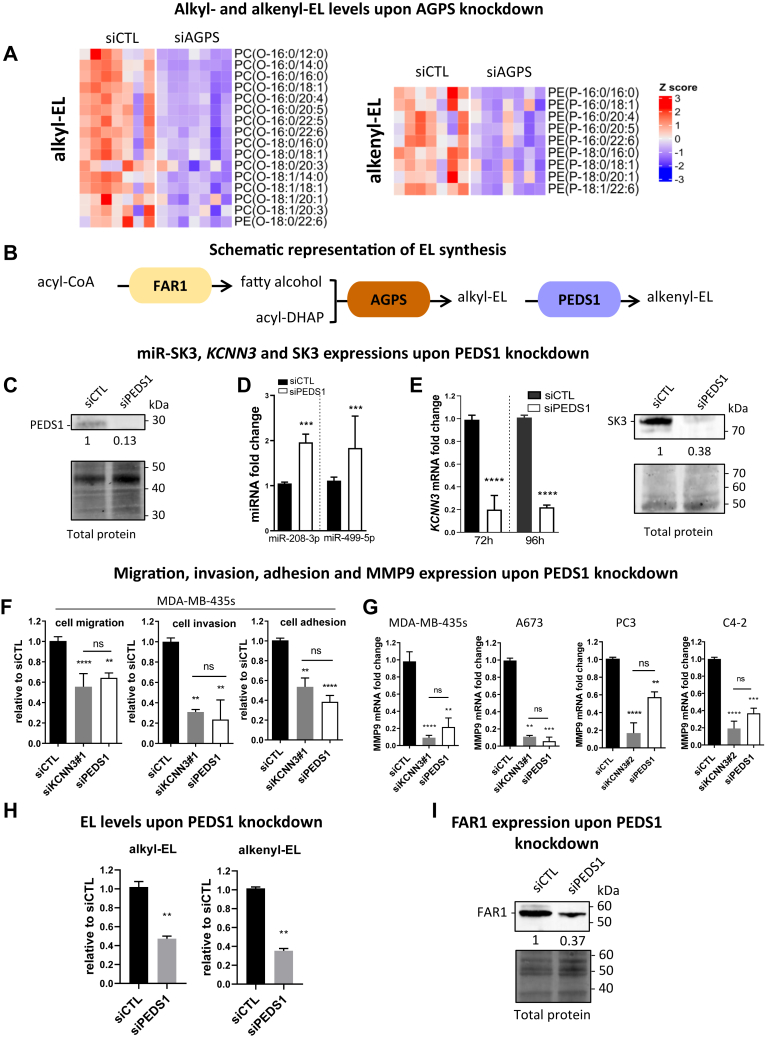
PEDS1, the key enzyme producing alkenyl-EL, is involved in the AGPS/SK3/MMP9 axis mediating cell migration, invasion, and adhesion. A: AGPS knockdown reduces all EL level in cancer cells. MDA-MB-435s cells were transfected with siCTL or siAGPS#1 during 96 h before lipid extraction. The heatmap represents the significantly altered molecular species of EL identified by UHPLC-MS in negative ion mode in the siCTL (left) and siAGPS (right) samples (see Supplemental Figs. S10 and S11 for the statistical tests performed for the selection, N = 7, Wilcoxon signed-rank test, ∗*P* < 0.05). Each column represents a different sample with siCTL and siAGPS samples working in pairs. Each line represents a different EL (identified on the right) and each colored square represents the Z-score of the EL (see material and methods for details on the Z-scores calculations). High Z-scores (red) indicate a higher quantity of EL in the sample and low Z-scores (blue) a lower quantity. B: Schematic representation of EL synthesis. The fatty acid of acyl dihydroxyacetone phosphate (acyl-DHAP) is replaced by a fatty alcohol produced by fatty acyl-CoA reductase 1 (FAR1) forming alkyl-DHAP in a reaction catalyzed by alkylglycerone phosphate synthase (AGPS). Alkyl-DHAP is catalyzed by several enzymes to form diverse alkyl-EL species. From alkyl-PE, plasmanylethanolamine desaturase 1 (PEDS1) forms alkenyl-PE and other enzymes enable the formation of other alkenyl-EL species. C–E: PEDS1 knockdown increases miRNAs targeting KCNN3 mRNA and reduces KCNN3 mRNA and SK3 expression. D: PEDS1 protein level studied in stain-free Western blot is highly decreased 72 h after transfection with siPEDS1 compared to siCTL in MDA-MB-435s cells. PEDS1 detection band area was normalized to the total protein signal of the lane and then relativized to the siCTL condition (N = 3). E: miR-499a-5p and miR-208-3p were quantified by RT-qPCR 48 h after transfection with siPEDS1 in MDA-MB-435s cells (N = 4). F: Left, *KCNN3* mRNA was quantified by RT-qPCR 72 and 96 h after transfection with siPEDS1 in MDA-MB-435s cells (N = 3) (median ± interquartile range, Mann–Whitney test, ∗∗∗∗*P* < 0.001). Right: SK3 protein level was measured by stain-free Western blot 72 h after transfection with siPEDS1 compared to siCTL in MDA-MB-435s cells. SK3 detection band area was normalized to the total protein signal of the lane and then relativized to the siCTL condition (N = 3). F: PEDS1 knockdown reduces cancer cell migration, invasion, and adhesion. MDA-MB-435s cells were transfected with siCTL, siKCNN3#1, or siPEDS1 during 72 h before migration, invasion, and adhesion assays. Data are relativised to siCTL condition (N = 3–4, median ± interquartile range, Kruskal–Wallis test, *P* < 0.0001 and post hoc Dunn’s test, compared to control condition: ∗∗∗∗*P* < 0.0001, ∗∗∗*P* < 0.001, ∗∗*P* < 0.01, and ns = not significant). G: *MMP9* expression is under the control of PEDS1 and SK3 expressions. *MMP9* mRNA was quantified by RT-qPCR 72 h after transfection with siCTL, siKCNN3#1, or siPEDS1 and relativised to siCTL condition (N = 3, median ± interquartile range, Kruskal–Wallis test, *P* < 0.0001 and post hoc Dunn’s test, compared to control condition: ∗∗*P* < 0.01, ∗∗∗*P* < 0.001, ∗∗∗∗*P* < 0.0001, and ns, not significant). H and I: PEDS1 knockdown also reduces the levels of all EL, possibly through the regulation of FAR1, the rate-limiting enzyme of EL synthesis. H: MDA-MB-435s cells were transfected with siCTL or siPEDS1 during 72 h before UHPLC-MS experiments in positive ion mode. Six independent experiments were performed. The fold changes plotted were calculated by relativizing the total abundances of alkyl-EL (left) and alkenyl-EL (right) in each siPEDS1 sample to their total abundance in the matching siCTL samples. (N = 6, median ± interquartile range, Mann–Whitney test, ∗∗*P* < 0.01). I: FAR1 protein level in MDA-MB-435s was measured by stain-free Western blot 72 h after transfection with siPEDS1 compared to siCTL. FAR1 detection band area was normalized to the total protein signal of the lane and then relativized to the siCTL condition (N = 3). DHAP, dihydroxyacetone phosphate; EL, ether lipid; miRNA, micro RNA; MMP, matrix metalloproteinase; qPCR, quantitiative polymerase chain reaction; RT-qPCR, reverse transcription quantitiative polymerase chain reaction; siCTL, si control; UHPLC-MS, ultra-high performance liquid chromatography tandem mass spectrometry.

The obvious mechanism through which AGPS controls SK3 expression would be through ELs, which are produced by AGPS as alkyl- and alkenyl-EL. We performed a targeted lipidomic analysis of siAGPS compared to si control cells using a LC-MS/MS–based protocol that enabled the identification of alkyl- and alkenyl-EL molecular species and their relative quantification. As shown in Fig. 4A and in the Supplemental Figs. S10 and S11, in negative ion mode, 35 molecular species of EL were identified, including alkyl- and alkenyl-EL (plasmalogens); among these, 25 were significantly reduced while the remaining showed the same tendency. The same was observed in positive ion mode, with the identification of a total of roughly 60 different EL species in our cells accounting for a broad overview of the EL cellular levels (see individual graphics in Supplemental Figs. S10–S14). Considering that the production of both alkyl- and alkenyl-EL was reduced when suppressing AGPS, we subsequently investigated the specific role of alkenyl-EL in the regulation of miR-208 and miR-499 targeting *KCNN3* by using a siRNA directed against PEDS1, the enzyme responsible for the desaturation of alkyl-EL into alkenyl-EL, encoded by *TMEM189* (see schematic representation in Fig. 4B). The delivery of siPEDS1 into cells inhibited the mRNA *TMEM189* encoding for PEDS1 by more than 90% (Supplemental Fig. S15) and the protein PEDS1 was inhibited by 87% 72 h after transfection (Fig. 4C). Interestingly, PEDS1 knockdown resulted in an increase of the identified miR-208 and miR-499 (Fig. 4D) similar to what was observed upon AGPS knockdown (Fig. 1F). In a similar way, PEDS1 knockdown induced a large reduction of *KCNN3* mRNA and SK3 protein quantities in various cell lines (Fig. 4E, Supplemental Fig. S16), alongside a decrease of SK3-dependent cell migration, invasion, and adhesion (Fig. 4F); the knockdown also almost totally abolished the expression of *MMP9* (Fig. 4G) as was observed following SK3 and AGPS knockdown (see Figs. 2 and 3). However, knocking down PEDS1 induced not only a reduction of alkenyl-EL quantities but also of alkyl-EL (Fig. 4H, Supplemental Figs. S17 and S18). This is surprising since an accumulation of alkyl-EL in PEDS1-deficient mice has been shown along with a decrease of alkenyl-EL (10). This could be explained by the downregulation of the fatty acyl-coA reductase 1 expression we observed (Fig. 4I), the rate-limiting enzyme of EL synthesis, as it reduces fatty acids into fatty alcohols which are then incorporated at the *sn*-1 position of the EL (Fig. 4B). Altogether, these results highlight a key role of alkyl- and alkenyl-EL in SK3 expression. Unfortunately, the knockdown of PEDS1 did not allow us to discriminate between the effects of alkyl-EL and alkenyl-EL on SK3.

### Alkyl- and alkenyl-EL supplementation promotes SK3 expression while only alkyl-EL increase SK3 activity

**Fig. 5:**
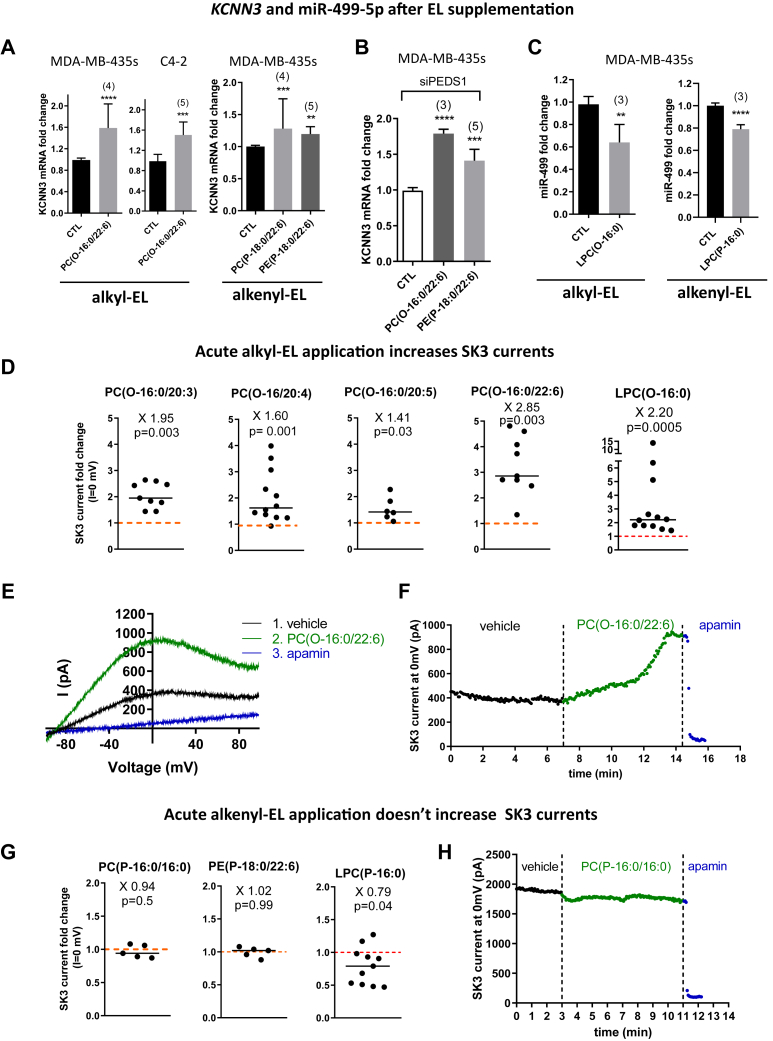
Alkyl- and alkenyl-EL promote *KCNN3* expression while only alkyl-EL increases SK3 channel activity. A and B: Supplementation with both alkyl- and alkenyl-EL increases KCNN3 expression and is able to restore partially its expression reduced after PEDS1 knockdown. Cells were treated daily with 20 μM of EL in liposomes for 96 h. Cells to be transfected were pretreated with EL liposomes for 24 h before transfection with siCTL or siPEDS1. After 6 h, the transfected cells were treated daily during the remaining 72 h. KCNN3 mRNA level measured by RT-qPCR is increased after supplementation with EL (median ± interquartile range, Mann–Whitney test, ∗∗∗∗*P* < 0.0001, ∗∗∗*P* < 0.001, and ∗*P* < 0.5). C: Expression of miR-499a-5p–targeting *KCNN3* mRNA is reduced after lyso-alkyl-EL and lyso-alkenyl-EL supplementation. MDA-MB-435s cells were treated daily with 20 μM of LPC(O-16:0) (left) and LPC(P-16:0) (right) in liposomes for 48 h, after which miR-499a-5p was quantified by RT-qPCR. D–H: Acute application of EL on SK3 currents. Whole-cell currents in HEK293T cells expressing recombinant human SK3 were generated by a ramp protocol from −100 to 100 mV in 500 ms from a constant holding of 0 mV with a pCa 6. D: Graphs representing the current recorded at 0 mV after acute application of alkyl-EL (3 μM). Data are relativised to the current recorded before alkyl-EL application. The black line indicates the median, each point represents SK3 current fold change after alkyl-EL application to one cell; bars, median. Wilcoxon signed-rank test. E: representative whole-cell currents recorded after application of vehicle (black trace, 7 min), after application to PC(O-16:0/22:6) (green trace, 3 μM, 7 min) and after addition of apamin (blue trace, 100 nM, less than 2 min) to inhibit SK3 currents. F: Graphs showing a representative time course at 0 mV of whole-cell currents (same cell than in E). G: Graphs representing the current recorded at 0 mV after acute application of alkenyl-EL (3 μM). Data are relativised to the current recorded before alkenyl-EL application. The black line indicates the median, each point represents SK3 current fold change after alkenyl-EL application to one cell; bars, median. Wilcoxon signed-rank test. H: Graphs showing a representative time course at 0 mV of whole-cell currents during application of vehicle, PC(P-16:0/C16:0), and apamin. AGPS, alkylglycerone phosphate synthase; EL, ether lipid; LPC, lysophosphatidylcholine; miRNA, micro RNA; PC, phosphatidylcholine; PEDS1, plasmanylethanolamine desaturase 1; qPCR, quantitiative polymerase chain reaction; RT-qPCR, reverse transcription quantitiative polymerase chain reaction; siCTL, si control.

To further study the role of alkyl- and alkenyl-EL produced by AGPS and PEDS1 on SK3 expression and activity, we supplemented our cells with commercially available EL in their phospholipid form, as identified by LC-MS in our cells (Fig. 4A, Supplemental Figs. S10–S14 and S17 and S18). We focused on the alkyl-EL PC(O-16:0/22:6) and the alkenyl-EL PE(P-18:0/22:6) as they were both identified in our cells (Fig. 4A for the alkyl-EL and Supplemental Fig. S12 for the alkenyl-EL). These specific EL species were chosen as they contain comparable *sn*-2 fatty acids, unfortunately, we could not chose an alkyl- and alkenyl-EL with comparable *sn*-1 fatty alcohol chains as they were none commercially available. In addition, although this EL specie was not identified in our cells, we also supplemented them with the alkenyl-EL PC(P-18:0/22:6) to be able to compare potential effects due to the difference of polar headgroup. As observed, both alkyl- and alkenyl-ELs (PC and PE) increased *KCNN3* mRNA (Fig. 5A). Since alkenyl-EL cannot be transformed back into alkyl-EL (Fig. 4C), this at least suggests that alkenyl-EL could promote *KCNN3* expression independently of their polar head. To verify whether alkyl-EL play the same role as alkenyl-EL, alkyl- and alkenyl-EL were then supplemented to cells after PEDS1 knockdown, preventing the metabolization of alkyl-EL into alkenyl-EL. Interestingly, both were able to increase *KCNN3* transcript expression similarly (Fig. 5B), confirming that both alkyl- and alkenyl-EL promoted *KCNN3* expression. Considering that both EL subfamilies were able to promote *KCNN3* expression, we next chose to supplement cells with the lyso-alkyl-EL lysophosphatidylcholine (LPC)(O-16:0) as it should be metabolized into the corresponding C16 alkyl- and alkenyl-EL with a variety of *sn*-2 fatty acids chains, and with the lyso-alkenyl-EL LPC(P-16:0) as it should be metabolized only into C16 alkenyl-EL. In addition, the previously supplemented EL could already be metabolized by phospholipase A2 (PLA2) into similar lyso-EL. Supplemental Fig. S19 in the Supporting information confirms that LPC(O-16:0) increased *KCNN3* transcript expression. In addition, both LPC(O-16:0) and LPC(P-16:0) supplementations reduced miR-499 expression (Fig. 5C), demonstrating the capacities of lyso-EL to promote SK3 expression by regulating miR expression. To further investigate the role of these lipids, we tested the effects of alkyl- and alkenyl-EL on SK3 activity using the patch-clamp technique to measure SK3 currents, following the acute application of individual ELs. Figure 5D shows that all six alkyl-ELs tested, including the lyso-EL LPC(O-16:0), increased the amplitude of SK3 currents with same efficiency since we observed no significant differences between different alkyl-EL species. The effect of alkyl-EL on SK3 currents was observed within 1 min (Fig. 5E) and 50% of the effect was reached 2–3 min after the alkyl-EL application, that is, longer than what is observed for ohmline (12). This effect appeared to be specific to alkyl-EL since the EL PC(16:0/20:4), the exact analog of PC(O-16:0/20:4), had no effect on SK3 currents (Supplemental Fig. S19). We then tested the effect of alkenyl-EL and found no effect on SK3 currents, demonstrating a differential role of alkyl- and alkenyl-EL on SK3 activity (Fig. 5G,H). Surprisingly, LPC(P-16:0) decreased 21% of the amplitude of SK3 current (Fig. 5G) in contrast to the corresponding alkyl LPC(O-16:0) (Fig. 5D) that was found to increase SK3 current by 2-fold.

## Discussion

**Fig. 6:**
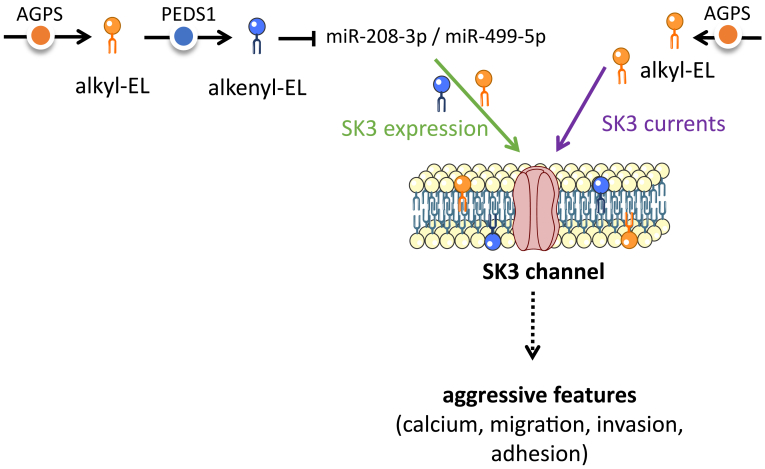
Schematic overview of SK3 regulation by EL. Both alkyl- and alkenyl-EL, requiring AGPS and PEDS1 for their endogenous synthesis, promote SK3 expression, through the downregulation of miRNAs targeting *KCNN3* transcripts (miR-SK3). Alkyl-EL, and not alkenyl-EL, also promotes SK3 currents. This could lead to a fine regulation of SK3-dependent biological functions involved in calcium entry, cell migration, invasion, and adhesion, thus promoting aggressive features of cancer cells. AGPS, alkylglycerone phosphate synthase; EL, ether lipid; miRNA, micro RNA; PEDS1, plasmanylethanolamine desaturase 1.

This study has demonstrated that in addition to being sensitive to synthetic alkyl-EL, SK3 channel is regulated by endogenous alkyl- and alkenyl-EL. The activity of SK3 channels (SK3 currents) was found to be either activated or inhibited by synthetic alkyl-EL (2), leading to the modulation of calcium entries and cell migration of various cancer cells (1, 24, 34, 35). In contrast, no regulation of SK3 expression by synthetic alkyl-EL was observed (36). Here, as summarized in Fig. 6, we found that silencing AGPS or PEDS1 decreased the expression of SK3 and its biological activities promoting the tumor aggressiveness of various cancer cells (calcium entry, MMP9 expression, migration, invasion, and adhesion). Since silencing AGPS reduced the quantity of alkyl- and alkenyl-ELs, and since the supplementation with both EL species promoted *KCNN3* expression, part of the effects of AGPS or PEDS1 on SK3 expression was likely due to the alkyl- and alkenyl-EL. Both alkyl- and alkenyl-EL supplementations were found to increase the *KCNN3* transcript expressions previously reduced by PEDS1 knockdown in a similar manner, confirming the role of both EL subfamilies independently.

Noteworthily, the effect of the well-known alkyl-EL platelet-activating factor (PAF) with 2 carbons *sn*-2 acyl chain was not studied in this system to avoid any effect simply due to PAF receptor or platelet activating factor receptor–mediated signaling. It can however be hypothesized that PAF could also promote *KCNN3* expression, since the *sn*-2 chain does not seem to be important in this regulation. In addition, following AGPS and PEDS1 knockdown, an increase of acyl-phospholipid species (ester-linked phospholipids) was observed as shown in Supplemental Fig. S22 and also already described (7). This increase of ELs leads us to hypothesize that rather than fluxing the precursors of EL to glycolytic pathway, the rewiring likely happens right before the AGPS step. More precisely, acyl-dihydroxyacetone phosphate could be directly converted to acyl-lysophosphatidic acid by the PexRAP enzyme for further metabolization into acyl-phospholipids rather than being converted to alkyl-lysophosphatidic acid. Since the EL supplemented cannot be metabolized into acyl-phospholipids, acyl-phospholipids are not responsible of the regulation of *KCNN3* transcripts expression and we showed that they had no effect on SK3 activity. The effect of this increase of acyl-phospholipids, which could be to balance out the decrease of EL, would however be interesting to explore, as it could have important biophysical consequences independently from the effect of EL on SK3.

The mechanism through which alkyl- and alkenyl-EL increase the expression of SK3 is unknown but depends on the negative regulation of miR-499 and miR-208. Although miRNA, including miR-499 (37), are known to regulate lipid metabolism (38), to our knowledge, the regulation of miRNA by lipids and alkyl- and alkenyl-EL has not been described. miRNA expression can be regulated either by transcriptional and posttranscriptional mechanisms, as well as by hypoxia or hormones such as oestrogen (39). Interestingly, promoter hypermethylation has been shown to be one of the major mechanisms for silencing miR-31 in triple-negative breast cancer cell lines (40). In addition, EL could regulate transcriptional miRNA expression through different mechanisms, such as i) a direct activation of the transcriptional factor PPAR© as demonstrated for alkyl-EL (41, 42) or indirectly by polyunsaturated fatty acids hydrolyzed from the *sn-2* position of EL following PLA2 activation (43), ii) through ligands of G protein-coupled receptors such as lysophosphatidic acid receptors which activate various signaling pathways and transcriptional factors (44), and iii) by modulating the biophysical properties of plasma membranes as constituents of lipid rafts nanodomains controlling cellular signaling (45, 46). The exact mechanism by which EL regulate the expression of miRNA should be explored.

It is tempting to speculate that if the synthesis of alkyl- and alkenyl-ELs increases during tumor development and progression, as initially shown by Snyder and Wood (47), this would increase the expression of SK3 and the development of metastases. This is in line with a study showing that AGPS is overexpressed in breast cancer tissues compared to noncancerous tissues (7) as well as with our data showing that the expressions of SK3 and AGPS is correlated in human breast tissues. Thus, an increase in the expression of AGPS and/or PEDS1 would increase alkyl- and alkenyl-EL production, SK3 expression, and the formation of SK3–Orai1 complexes, thus leading to CCE, cell migration, and the development of bone metastasis (1). It is not surprising that AGPS was expressed in these aggressive cell lines (MDA-MB-435s, PC3, A-673, C4-2) as Benjamin *et al.* (2013) showed that this enzyme is overexpressed in various aggressive cancer cell lines, including in the prostate, breast, and melanomas. Interestingly, we found a positive correlation between SK3 and AGPS expression in tissues other than tumorous ones (Supplemental Fig. S8A) such as the brain and heart, where SK3 has been found to control excitation-secretion/contraction coupling (48, 49). This suggests that alkyl- and alkenyl-EL could control the expression of SK3 within these organs and could explain part of the neurodegenerative and cardiovascular diseases observed when EL quantities are decreased in these pathologies (5). In parallel, this study on EL revealed the existence of a new and unknown function for SK3, namely a nonpore function that promotes cell adhesion and invasion through AGPS and MMP9 expression. This nonconducting function of SK3 could be explained by its gating currents induced by its conformational changes such as those observed for EAG-1 and Kv1.3 channels, leading to the activation of partner enzymes such as p38 MAPK and Ca2+/calmodulin-dependent protein kinase II (50, 51, 52) and/or by physical interactions with kinases such as those observed with intermediate calcium-activated potassium channel that promote cell proliferation by regulating the extracellular signal-regulated kinases 1/2 and JNK signaling pathways. Furthermore, and more related to adhesion and invasion processes, SK3 could interact with transmembrane proteins that facilitate cell-cell and cell-extracellular matrix interactions such as α5 integrin, as already observed for Kv2.1 (53). Further research is needed to explore this new function of SK3 regulated by EL.

Of note, silencing PEDS1 in this study resulted in an unexpected outcome as alkyl-ELs were also reduced. While we were able to show that this was due to a downregulation of fatty acyl-coA reductase 1 expression, this result remains contradictory to what is described in the literature. This contradiction could be due to the fact PEDS1 silencing was only transitory in our study compared to the stable extinctions described in the literature (10), which could lead to different compensations.

With regard to the canonical function of SK3, its channel function and the production of a potassium current, we observed that all tested alkyl-ELs, including lyso-alkyl-EL, with some also promoting SK3 expression (PC(O-16/22:6), LPC(O-16:0)), increased the amplitude of SK3 currents. This effect was not observed with the EL PC(16:0/20:4) when compared to its alkyl-EL analog PC(O-16:0/20:4), suggesting that the *sn-*1 ether bond plays an essential role in alkyl-EL effect on SK3 activity. The essential role of the ether bond on SK3 has already been observed with the synthetic alkyl-EL ohmline or with the well-studied endogenous alkyl-EL PAF (2). The acute effect of these lipids on SK3 currents could not be explained by the inverted conical shape of the lipids as observed for TREK/TRAAK channels (54) because alkyl-EL (which have a nonconical shape) and lyso-alkyl-EL (inverted conical shape) both increase SK3 activity. We believe that alkyl-EL may act on SK3 channels by modifying the biophysical properties of their membranes, as already observed with the synthetic-alkyl-EL ohmline that induced a reorganization of the lipid domains of membranes (46). Nevertheless, we cannot exclude other mechanisms such as specific lipid-protein interactions as observed for intermediate calcium-activated potassium channel (55).

Interestingly, unlike alkyl-EL, alkenyl-ELs had no effect or decreased (for the lyso alkenyl-EL) SK3 currents. The differential effects of alkyl- and alkenyl-ELs could be explained by their different biophysical properties when inserted into a membrane, as the vinyl-ether bond would increase the membrane thickness (56) compared to an ether bond; alternatively, this difference could be explained by the antioxidant role of alkenyl-EL, which is not observed for alkyl-EL (57). Moreover, the fact that lyso-alkyl-ELs could retain their effects suggests that alkyl-ELs could still exert their effect on SK3 activity as well as on its expression in pathophysiological conditions, where PLA2 is activated such as hypoxia, ischemia, and inflammation; indeed, our results suggest that LPC(O-16:0) was also able to promote SK3 expression. This would be particularly important in neurodegenerative and cardiovascular diseases, where EL quantities are reduced and associated with an increased excitability. Maintaining the SK3 activating activity of lyso-alkyl-EL could limit the severity of the disease. In contrast, the reduction of SK3 activity by lyso-alkenyl-EL could increase the severity of the pathology by increasing nervous and cardiac excitabilities. Future studies on EL should therefore focus not only on alkenyl-EL (as is the case today) but also on alkyl-EL.

It would also be of major interest to conduct a comprehensive study on the amount of alkyl-EL and of alkenyl-EL in benign and malignant tumorous tissues from various cancers compared to adjacent nontumorous tissues and corresponding tissues from healthy donors, as the existing studies performed somewhat differ in terms of both results and experimental plans. Undertaking such a thorough study could contribute reaching a consensus on the variation of EL in cancer and could provide more insights into the specific roles played by alkyl and alkenyl-EL in the pathogenesis of this pathology.

Moreover, in addition to cancer, variations of EL production may occur in neurodegenerative and cardiovascular diseases, leading to the dysfunction of excitation-coupling responses. These results could be used to develop therapies aiming to address ion channel dysfunction by modifying alkyl- and alkenyl-EL compositions. In addition, alkyl/alkenyl-EL, some of which belong to a new class of SK3 modulators, could be used in clinical applications as inhibitors of bone metastases and the peripheral neuropathic effect of chemotherapeutic agents in cancer or for specific neurodegenerative and cardiovascular diseases.

## Data Availability

All data needed to evaluate the conclusions of the paper are present in the main text and Supporting information. Therefore, all data are readily available to be shared. There are no exceptions to the sharing of data, materials, and softwares.

## Supplemental data

This article contains supplemental data.

## Conflict of interest

The authors declare that they have no conflict of interests with the contents of article.
