# Supplementary material for: Endogenous ether lipids differentially promote tumor aggressiveness by regulating the SK3 channel
Source: J Lipid Res. 2024 Apr 18;65(5):100544. doi: 10.1016/j.jlr.2024.100544 (PMC11127165; doi:10.1016/j.jlr.2024.100544)
Supplement: Supplemental Data [file mmc1.docx]

**Supplemental Text 1: APPENDIX METHODS**

**Cell viability assay:**

Cell proliferation was assessed using the MTT method. Cells were seeded into 24-wells plates and cell proliferation assay was performed at several times after siRNA transfection (24 hours to 96 hours) by addition of 400 µL of tetrazolium salt incubated for 45 minutes at 37° C. Reduced salt was solubilized in DMSO and then absorbance was measured at 570 nm.

**Store-operated calcium entry measurements:**

Cells were seeded into a 96-wells plate at 40 000 cells per well 48 hour after transfection with siRNA, the day before measurement. Fura-2 AM was loaded at 1 µM for 45 minutes at 37°C. Then medium was removed and 100 µL of calcium-free physiological saline solution per well was added. Measurements were performed with FlexStation (Molecular Device) at 510 nm after excitation at 340 and 380 nm. After 100 seconds of reading, 4 µM of thapsigargin (Molecular Probes) was added to deplete calcium from the ER. At 500 seconds 2 mM of calcium was added. The ratio 340/380nm is proportional to intracellular calcium concentration.

**Mass spectrometry:**

LC-HRMS analysis was performed on a UHPLC Ultimate 3000 system (Dionex, Sunnyvale, CA), coupled to a Q-Exactive mass spectrometer (Thermo Fisher Scientific) and operated in positive ionization mode (ESI+). Chromatography was carried out with a 1.7 μm XB—C18 (150 mm × 2.10 mm, 100 ̊A) UH- PLC column (Kinetex, Phenomenex, Torrance, CA) heated at 55◦C. The solvent system comprised mo- bile phase A [isopropanol/ACN (9:1) + 0.1% (vol/vol) formic acid + 10 mM ammonium formate], and mobile phase B [ACN/water (6:1) + 0.1% (vol/vol) formic acid + 10 mM ammonium formate]; the gradient operated at a flow rate of 0.26 mL/min over a run time of 24 min. The multisteps gradient was programmed as follows: 0 to1.5min,32%to45%A;1.5to5min,45%to52% A;5to8min,52%to58%A;8to11min,58%to66% A;11to14min,66%to70%A;14to18min,70%to 75%A;18to21min,75%to97%A;21to24min,97% A. The autosampler temperature (Ultimate WPS-3000 UHPLC system, Dionex) was set at 4◦C, and the in- jection volume for each sample was 5 μL. The heated ESI source parameters were a spray voltage of 3.5 kV, capillary temperature of 350◦C, heater temperature of 250◦C, sheath gas flow of 35 arbitrary units (AU), auxiliary gas flow of 10 AU, spare gas flow of 1 AU, and tube lens voltage of 60 V for C18. During the full-scan acquisition, which ranged from 250 to 1600 m/z, the instrument operated at 70,000 resolution, with an automatic gain control target of 1 × 106 charges and a maximum injection time of 250 ms.

The instrumental stability was evaluated by multiple injections (n = 5) of a quality control (QC) sample obtained from a pool of 10 μL of all samples analyzed. This QC sample was injected once at the beginning of the analysis, between every 10 sample injections, and at the end of the run.

**Supplemental Table S1 :** Patients characteristics for the cohort 1. Tumors were classified according to their phenotype in immunohistochemistry (and/or CISH for HER2) as follow: Luminal A (ER and/or positive PR, low proliferation index <14% and HER2 negative), Luminal B (ER and/or positive PR, high proliferation index> 14% and/or HER2 positive), HER2 (negative hormone receptors and HER2 positive) and Triple negative (hormone receptors and HER2 negative).

| **Age y, median (range) :** | 56 (31-88) |
| --- | --- |
| **SBR grade (n) :** | |
| 1 | 0 |
| 2 | 37 |
| 3 | 41 |
| **Classification (n) :** | |
| Luminal A | 44 |
| Luminal B | 15 |
| Triple negative | 11 |
| HER2 | 8 |

**Supplemental Table S2 :** Patients characteristics for the cohort 2. Tumors were classified according to their phenotype in immunohistochemistry as described for the cohort1.

| **Age y, median (range) :** | 57 (36-88) |
| --- | --- |
| **SBR grade (n) :** | |
| 1 | 4 |
| 2 | 17 |
| 3 | 28 |
| unknown | 1 |
| **Classification (n) :** | |
| Luminal A | 14 |
| Luminal B | 16 |
| Triple negative | 11 |
| HER2 | 9 |

**Supplemental Table S3 :** Patients characteristics for the cohort 3. Tumors were classified according to their phenotype in immunohistochemistry as described for the cohort 1.

| **Age y, median (range) :** | 63 (39-88) |
| --- | --- |
| **SBR grade (n) :** | |
| 1 | 1 |
| 2 | 15 |
| 3 | 13 |
| **Classification (n) :** | |
| Luminal A | 21 (A+B) |
| Luminal B |  |
| Triple negative | 5 |
| HER2 | 3 |

**Supplemental Table S4 :** Lipids used. BHT: butylated hydroxytoluene, CHCl_3_: chloroform, EtOH: ethanol,

| **Lipid** | **Solvent, concentration of stock solution** | **Reference** |
| --- | --- | --- |
| PC(O-16:0/20:3) | 1mg/mL, EtOH+0.1%BHT | 60901, Cayman Chemical |
| PC(O-16:0/20:4) | 10mg/mL, EtOH+0.1%BHT | 60904, Cayman Chemical |
| PC(O-16:0/20:5) | 1mg/mL, EtOH+0.1%BHT | 60907, Cayman Chemical |
| PC(O-16:0/22:6) | 10mg/mL, EtOH+0.1%BHT | 60903, Cayman Chemical |
| LPC(O-16:0) | 10mg/mL, CHCl_3_ | 60906, Cayman Chemical |
| PC(P-16:0/16:0) | 10mg/mL, EtOH+0.1%BHT | 28348, Cayman Chemical |
| PC(P-18:0/22:6) | 1mg/mL, CHCl_3_ | 852472C, Sigma-Aldrich |
| PE(P-18:0/22:6) | 1mg/mL, CHCl_3_ | 852806C, Sigma-Aldrich |
| LPC(P-16:0) | 10mg/mL, CHCl_3_ | 852464P, Sigma-Aldrich |
| PC(16:0/20:4) | 10mg/mL, CHCl_3_ | 850459C, Sigma-Aldrich |

**Supplemental Table S5 :** Antibodies used for western blot (WB) and immunohistochemistry (IHC). ON : overnight, RT: room temperature.

| **Protein** | **WB condition** | **IHC condition** | **Reference** |
| --- | --- | --- | --- |
| SK3 | 1/500, ON, 4°C | 1/100, 1h, RT | AB5350, Merk |
| AGPS | 1/800, ON, 4°C | 1/100, 1h, RT | HPA 030211, Sigma prestige |
| FAR1 | 1/500, ON, 4°C |  | NBP1-89847, Novus Biologicals |
| PEDS1 | 1/500, ON, 4°C |  | PA597031, Invitrogen |

**Supplemental Table S6 :** siRNA sequences used for transfection assays

| Name | Sequence 5’-3’ | Refseq NM targeted gene |
| --- | --- | --- |
| siCTL | GCCGACCAAUUCACGGCCG | none |
| siAGPS#1 | GCGAAUUCCUGAUAUAGUU | [NM_003659.4](https://www.ncbi.nlm.nih.gov/entrez/viewer.fcgi?db=nucleotide&id=1519315989) |
| siAGPS#2 | UCAAGGACCUCGUAUGUCA | [NM_003659.4](https://www.ncbi.nlm.nih.gov/entrez/viewer.fcgi?db=nucleotide&id=1519315989) |
| siKCNN3#1 | UAGUCACUCAGUCGCUUUC | [NM_002249.6](https://www.ncbi.nlm.nih.gov/entrez/viewer.fcgi?db=nucleotide&id=1519242379)  [NM_001204087.2](https://www.ncbi.nlm.nih.gov/nucleotide/NM_001204087.2?report=genbank&log$=nuclalign&blast_rank=34&RID=E65KECXG013) |
| siKCNN3#2 | GACUUAAUCACAGAACUCA | [NM_170782.3](https://www.ncbi.nlm.nih.gov/nucleotide/NM_170782.3?report=genbank&log$=nucltop&blast_rank=1&RID=E6619AW1016)  [NM_002249.6](https://www.ncbi.nlm.nih.gov/entrez/viewer.fcgi?db=nucleotide&id=1519242379)   \|  \| [NM_001365837.1](https://www.ncbi.nlm.nih.gov/nucleotide/NM_001365837.1?report=genbank&log$=nucltop&blast_rank=3&RID=E6619AW1016) \| \| --- \| --- \|   [NM_001204087.2](https://www.ncbi.nlm.nih.gov/nucleotide/NM_001204087.2?report=genbank&log$=nucltop&blast_rank=5&RID=E6619AW1016)   \|  \| [NM_001365838.1](https://www.ncbi.nlm.nih.gov/nucleotide/NM_001365838.1?report=genbank&log$=nucltop&blast_rank=4&RID=E6619AW1016) \| \| --- \| --- \| |
| siPEDS1 | CACAUUGACCCGACAGCUA | [NM_170782.3](https://www.ncbi.nlm.nih.gov/nucleotide/NM_170782.3?report=genbank&log$=nucltop&blast_rank=1&RID=E6619AW1016)  [NM_002249.6](https://www.ncbi.nlm.nih.gov/entrez/viewer.fcgi?db=nucleotide&id=1519242379) |
| siMMP9 | CAUCACCUAUUGGAUCCAA | [NM_004994.3](https://www.ncbi.nlm.nih.gov/entrez/viewer.fcgi?db=nucleotide&id=1519311730) |

**Supplemental Table S7 :** Primers used for quantitative real-time PCR.

| gene | Forward 5’-3’ | Exon | Reverse3’- 5’ | Exon | Refseq NM |
| --- | --- | --- | --- | --- | --- |
| KCNN3 | TGGACACTCAGCTCACCAAG | 5 | GTTCCATCTTGACGCTCCTC | 7 | [NM_001204087.2](https://www.ncbi.nlm.nih.gov/entrez/viewer.fcgi?db=nucleotide&id=1890267156)  [NM_170782.3](https://www.ncbi.nlm.nih.gov/entrez/viewer.fcgi?db=nucleotide&id=1675077212)  [NM_002249.6](https://www.ncbi.nlm.nih.gov/entrez/viewer.fcgi?db=nucleotide&id=1519242379)  [NM_001365837.1](https://www.ncbi.nlm.nih.gov/entrez/viewer.fcgi?db=nucleotide&id=1475928933)  [NM_001365838.1](https://www.ncbi.nlm.nih.gov/entrez/viewer.fcgi?db=nucleotide&id=1475928783) |
| AGPS | CCCTTGGAGTAAATGTGGAGCA | 3 | TGGCATGTTGGCCATAAAACTA | 6 | [NM_003659.4](https://www.ncbi.nlm.nih.gov/entrez/viewer.fcgi?db=nucleotide&id=1519315989) |
| PEDS | TCACCCCACGAGACCTACTT | 5 | TTCATGTCATCTGCCCGAGG | 6 | [NM_170782.3](https://www.ncbi.nlm.nih.gov/nucleotide/NM_170782.3?report=genbank&log$=nucltop&blast_rank=1&RID=E6619AW1016)  [NM_002249.6](https://www.ncbi.nlm.nih.gov/entrez/viewer.fcgi?db=nucleotide&id=1519242379) |
| MMP9 | ACGCACGACGTCTTCCAGTA | 12 | \|  \| CCACCTGGTTCAACTCACTCC \| \| --- \| --- \| \|  \|  \| | 13 | [NM_004994.3](https://www.ncbi.nlm.nih.gov/entrez/viewer.fcgi?db=nucleotide&id=1519311730) |
| MMP2 | GATGATGCCTTTGCTCGTGC | 3 | CAAAGGGGTATCCATCGCCA | 4 | [NM_004530.6](https://www.ncbi.nlm.nih.gov/entrez/viewer.fcgi?db=nucleotide&id=1519242484)  [NM_001302510.2](https://www.ncbi.nlm.nih.gov/entrez/viewer.fcgi?db=nucleotide&id=1890269344)  [NM_001302509.2](https://www.ncbi.nlm.nih.gov/entrez/viewer.fcgi?db=nucleotide&id=1890263847)  [NM_001127891.3](https://www.ncbi.nlm.nih.gov/entrez/viewer.fcgi?db=nucleotide&id=1889590737)  [NM_001302508.1](https://www.ncbi.nlm.nih.gov/entrez/viewer.fcgi?db=nucleotide&id=700274110) |
| ORAI1 | AGGTGATGAGCCTCAACGAG | 1 | CTGATCATGAGCGCAAACAG | 2 | [NM_032790.3](https://www.ncbi.nlm.nih.gov/entrez/viewer.fcgi?db=nucleotide&id=170932551) |
| ZEB1 | TGCACTGAGTGTGGAAAAGC |  | TGGTGATGCTGAAAGAGACG |  |  |
| SNAIL1 | CCTGGGTGCCCTCAAGATG | 1 | CCGGACTCTTGGTGCTTGT | 3 | [NM_005985.4](https://www.ncbi.nlm.nih.gov/entrez/viewer.fcgi?db=nucleotide&id=1519243938) |
| TWIST | GCAGCTATGTGGCTCACGA | 1 | TCTCTGGAAACAATGACATCTAGG | 2 | [NM_000474.4](https://www.ncbi.nlm.nih.gov/entrez/viewer.fcgi?db=nucleotide&id=1519316069) |
| HPRT | TGACCTTGATTTATTTTGCATACC | 2 | CGAGCAAGACGTTCAGTCCT | 3 | NM_000194.3 |
| ALAS-1 | AGATCTGACCCCTCAGTCCC | 8 | TCCACGAAGGTGATTGCTCC | 9 | [NM_000688.5](https://www.ncbi.nlm.nih.gov/entrez/viewer.fcgi?db=nucleotide&id=362999011)  [NM_199166.2](https://www.ncbi.nlm.nih.gov/entrez/viewer.fcgi?db=nucleotide&id=362999012)  [NM_001304443.1](https://www.ncbi.nlm.nih.gov/entrez/viewer.fcgi?db=nucleotide&id=751130463)  [NM_001304444.1](https://www.ncbi.nlm.nih.gov/entrez/viewer.fcgi?db=nucleotide&id=751130482) |
| TBP | TGTATCCACAGTGAATCTTGGTTG | 4 | GGTTCGTGGCTCTCTTATCCTC | 5 | [NM_003194.5](https://www.ncbi.nlm.nih.gov/entrez/viewer.fcgi?db=nucleotide&id=1519313030)  [NM_001172085.2](https://www.ncbi.nlm.nih.gov/entrez/viewer.fcgi?db=nucleotide&id=1890284568) |

siCTL

siAGPS#1

AGPS

75 kDa

A)

B)

MDA-MB-435s

PC3

MDA-MB-435s

A673

1

0.27

MDA-MB-435s

E)


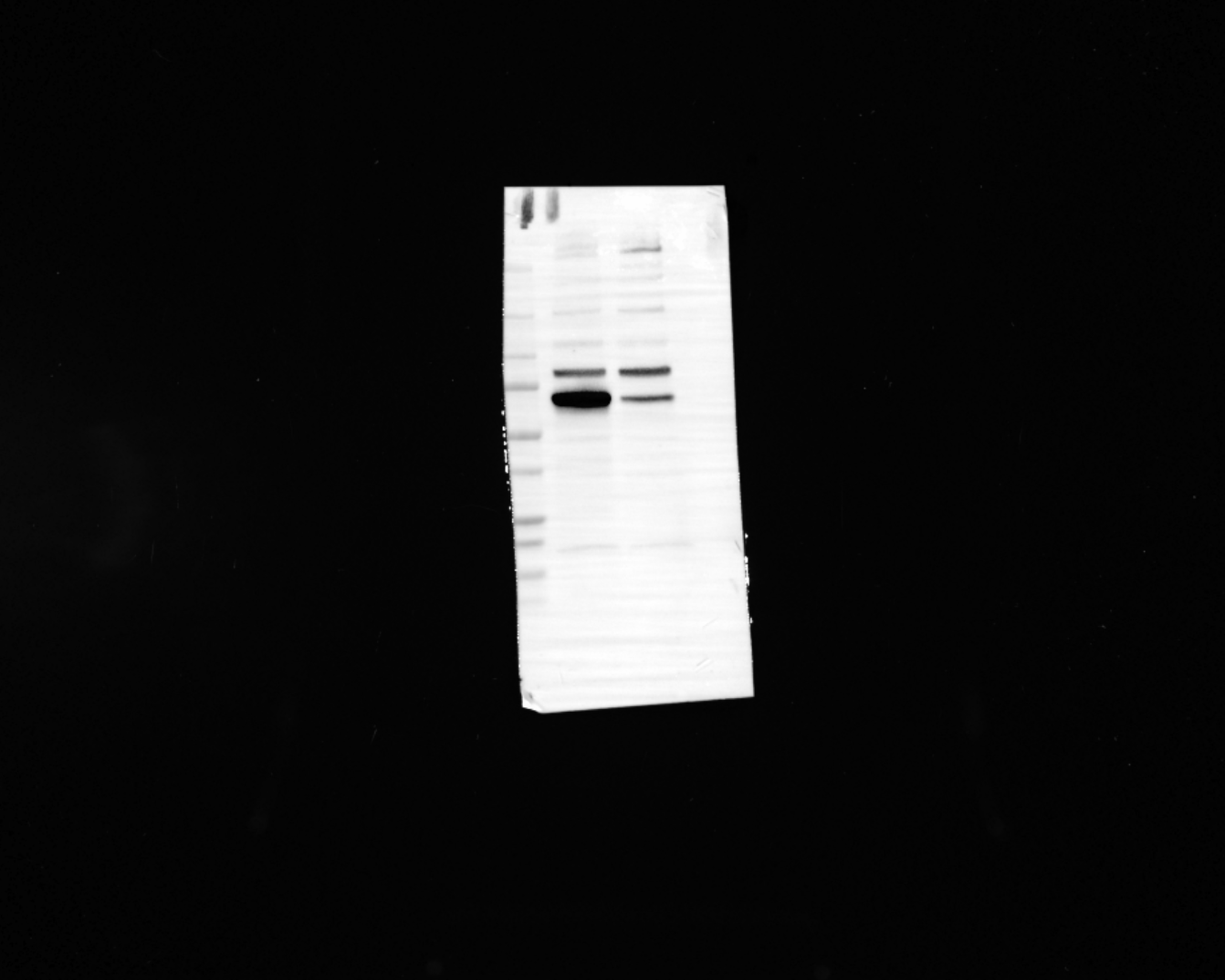

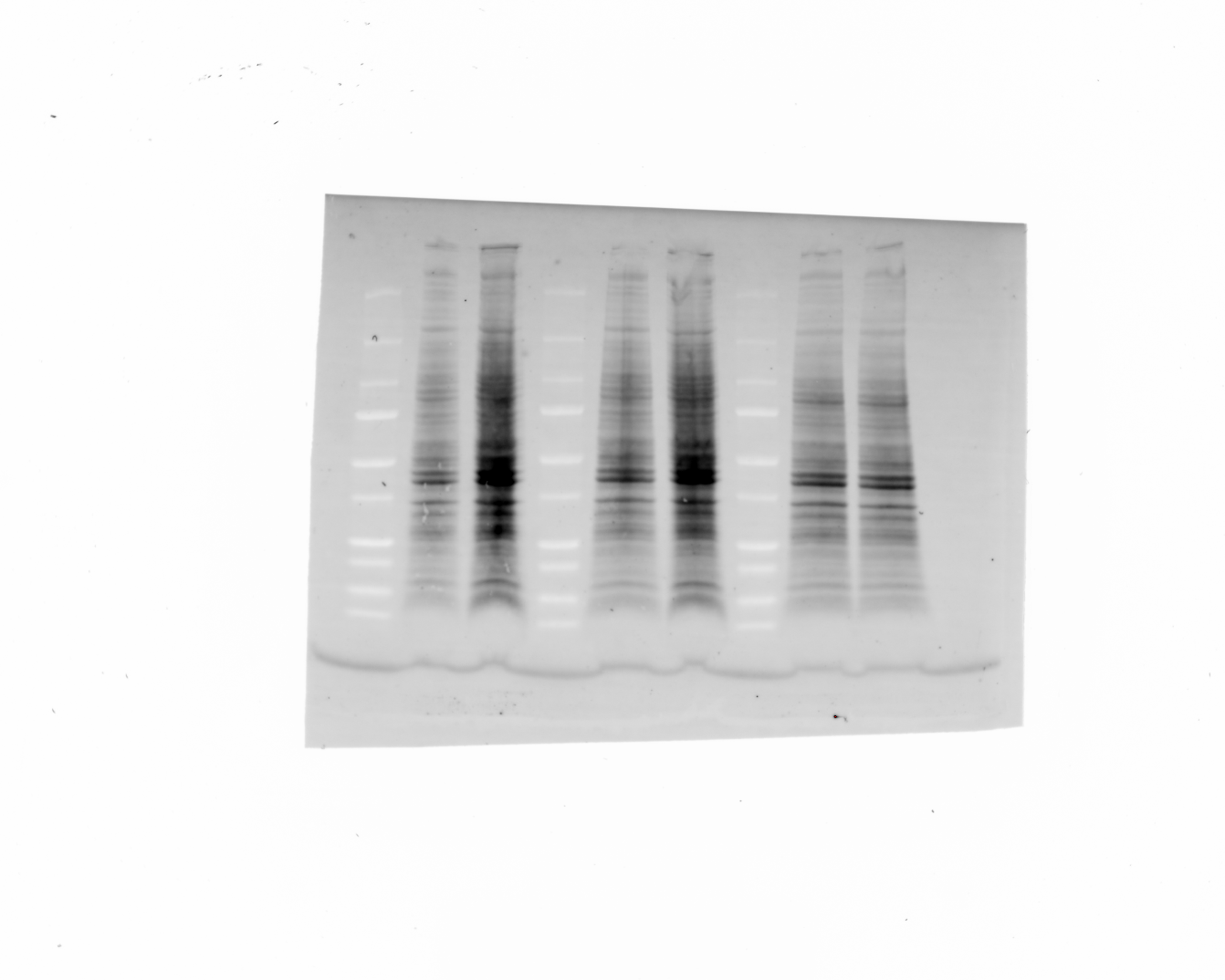


Total protein

C)

D)

**Supplemental Fig. S1. Validation of AGPS knockdown by siRNA.**

A, The protein expression level of AGPS decreased 72 hours after transfection with siAGPS in MDA-MB-435s cells (N=3). A representative blot is presented. Stain free technology was used for total protein normalization. The protein expression level of AGPS in the siAGPS#1 condition was relativized to siCTL and the mean value of the 3 experiments is displayed bellow the pictures. B-E, KCNN3 mRNA level analysed by RT-qPCR decreased after transfection with siAGPS#1 (B-D) or siAGPS#2 (E) in MDA-MB-435s, A673 and PC3 cell lines. Results are relativised to siCTL. Graphics showing median ± interquartile range, Mann-Whitney test, ****p<0.0001). The number in the brackets is the number of independent experiments performed.

B)

A)

**Supplemental Fig. S2. The level of KCNN3 mRNA decreases under AGPS knockdown in MDA-MB-435s cells.**

A, Kinetic of KCNN3 mRNA level after transfection with analysed by RT-qPCR transfection with siAGPS#1 B, KCNN3 mRNA level 72h after transfection with siAGPS#2 analysed by RT-QPCR. Results are relativised to siCTL. Graphics showing median ± interquartile range, Mann-whitney test, ****p<0.0001). The number in the brackets is the number of independent experiments performed.

**Supplemental Fig. S3. Zeb1, Snail and Twist expression are not affected under AGPS knockdown.** MDA-MB-435s and C4-2 cells were tranfected with siAGPS#1 during 72h before RT-qPCR experiments. Results are relativised to siCTL. Graphics showing median ± interquartile range, Mann-Whitney test, p>0,05. The number in the brackets is the number of independent experiments performed.

MDA-MB-435s

C)

MDA-MB-435s

B)

C4-2

MDA-MB-435s

A)

**Supplemental Fig. S4. Cell viability is few affected under AGPS knockdown.** MDA-MB-435s cells were tranfected with siAGPS#1 during 24, 48,72, and 96h before MTT assays. Results are relativised to siCTL for each kinetic points (N=3, median ± interquartile range, Mann-Whitney test, ns = no significant, * p>0,05, ***p>0,001).

A)

2 Ca

0 Ca

Tg

B)

**Supplemental Fig. S5. Store operated calcium entry (SOCE) and Orai1 mRNA is not affected by AGPS knock-down**

MDA-MB-435s cells were transfected during 72h with siAGPS#1 or siCTL. A, Calcium measurements were assessed by spectroflurometry with Fura-2 calcium probe. Left, Representative trace of SOCE. Middle, Thapsigargin (Tg) area which corresponds to area under curve of calcium release from ER. Right, 2Ca entry which corresponds to calcium entry amplitude. Data were relativized to results obtained with siCTL cells. (N=5, n=79, median ± interquartile range, Mann-Whitney test, ns = no significant). B, Expression of Orai1 mRNA by RT-qPCR experiments. Results are relativised to siCTL. Graphics showing median ± interquartile range, Mann-Whitney test, p>0,05, ns= no significant. The number in the brackets is the number of independent experiments performed.

PC3

A673

MDA-MB-435s

**Supplemental Fig. S6. Validation of SK3 knockdown by siRNA.**

MDA-MB-435s were transfected during 72h with SiKCNN3#1, SiKCNN3#2 or a siCTL and KCNN3 mRNA were analysed by RT-qPCR. Results are relativised to siCTL. Graphics showing median ± interquartile range, Mann-Whitney test, ****p<0.0001, N=3.

**Supplemental Fig. S7. Cell adhesion are under control of SK3 expression.** MDA-MB-435s cells were tranfected with siKCNN3#2 during 72h before cell adhesion assays. Results are relativised to siCTL (N=3, median ± interquartile range, Mann-Whitney test, **** p<0.0001).

MDA-MB-435s

B)

A)

MDA-MB-435s

**Supplemental Fig. S8. A, MMP2 expression are under the control of AGPS but not SK3.** MMP2 expression was assessed by RT-qPCR 72 hours after transfection with SiCTL (N=7), siAGPS#1 (N=7) or SiSK3#1(N=3), and relativised to SiCTL condition (median ± interquartile range, Kruskall wallis test, p<0.0001 and post hoc dunn’s test, compared to control condition **** p<0.0001, ns = no significant). B, **Validation of MMP9 knockdown by siRNA.** MDA-MB-435s were transfected with a siMMP9 or a siCTL and MMP9 mRNA were analysed by RT-qPCR 72h after. Results are relativised to siCTL. Graphics showing median ± interquartile range, Mann-Whitney test, ****p<0.0001, N=3.

**Supplemental Fig. S9. Apamin treatment increases SK3 and MMP9 expression.** A673 cells were treated daily for 3 days with apamin (100 nM) before RT-qPCR experiment. Results are relativised to siCTL (N=3, median ± interquartile range, Mann-Whitney test, **** p<0.0001, *p<0,05).

**Supplemental Fig. S10. The level of alkyl-EL species identified by UHLC-MS (negative mode) are reduced after AGPS knock-down.** MDA-MB-435s cells were transfected with siCTL or siAGPS#1 during 96h before UHPLC-MS experiments in negative ion mode. 7 independent experiments were performed and paired. (N=7; Points, N; bars, median, Wilcoxon signed-rank test, * p<0.05, ns = non significant.)

**Supplemental Fig. S11. The level of alkenyl-EL species identified by UHLC-MS (negative mode) are reduced after AGPS knock-down.** MDA-MB-435s cells were transfected with siCTL or siAGPS#1 during 96h before UHPLC-MS experiments in negative ion mode. 7 independent experiments were performed and paired. (N=7; Points, N; bars, median, Wilcoxon signed-rank test, * p<0.05, ns = non significant.)


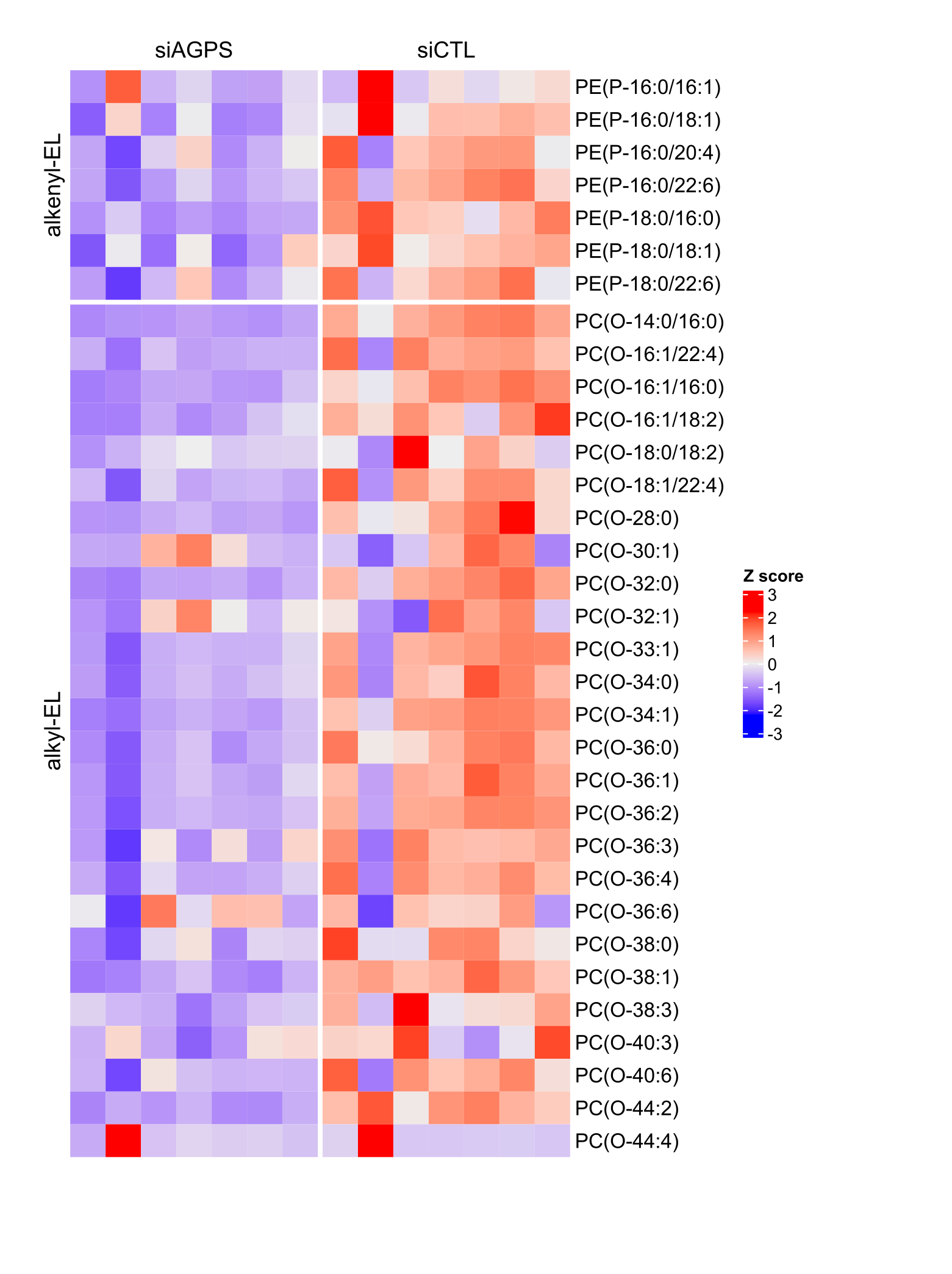

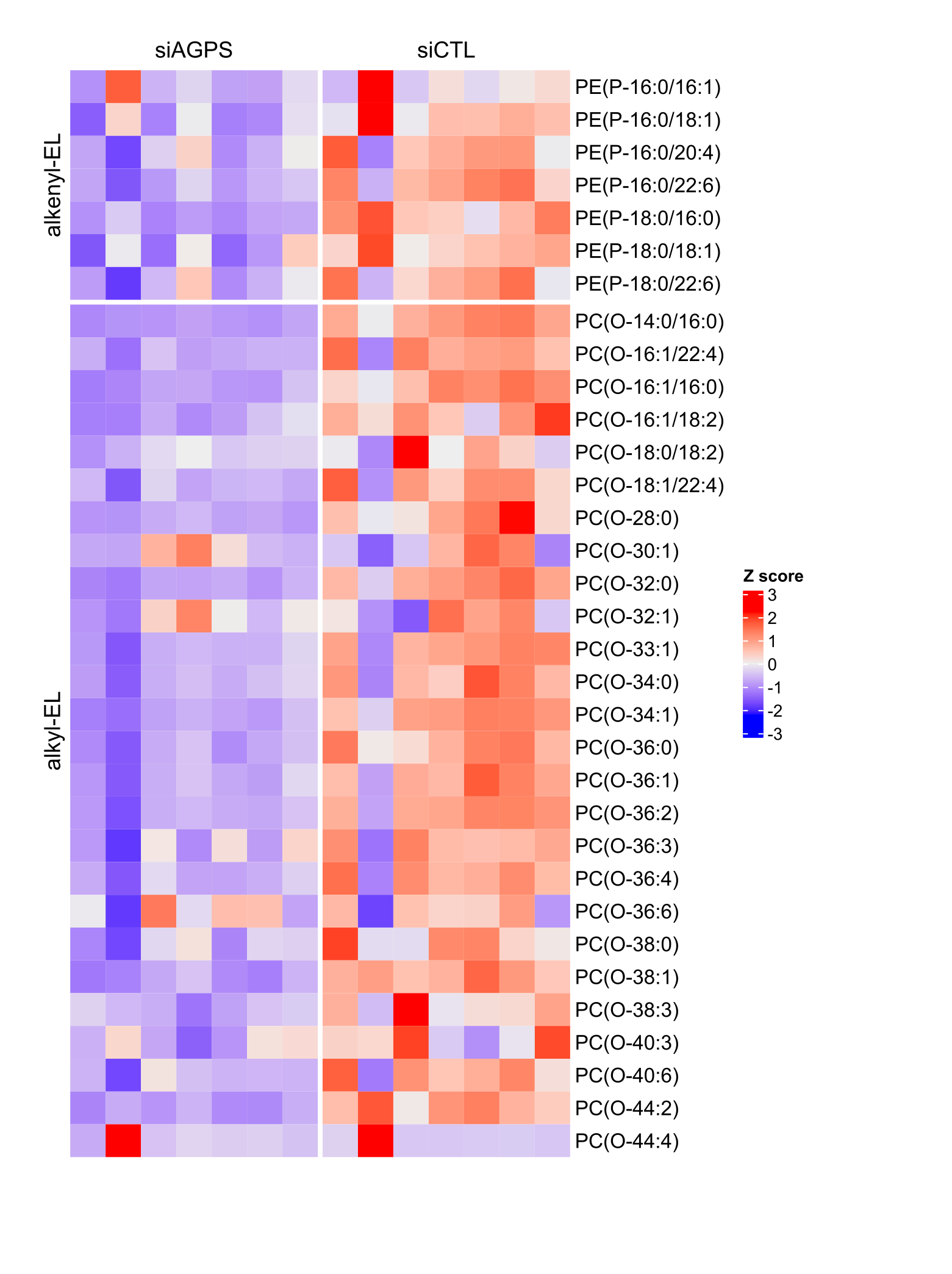

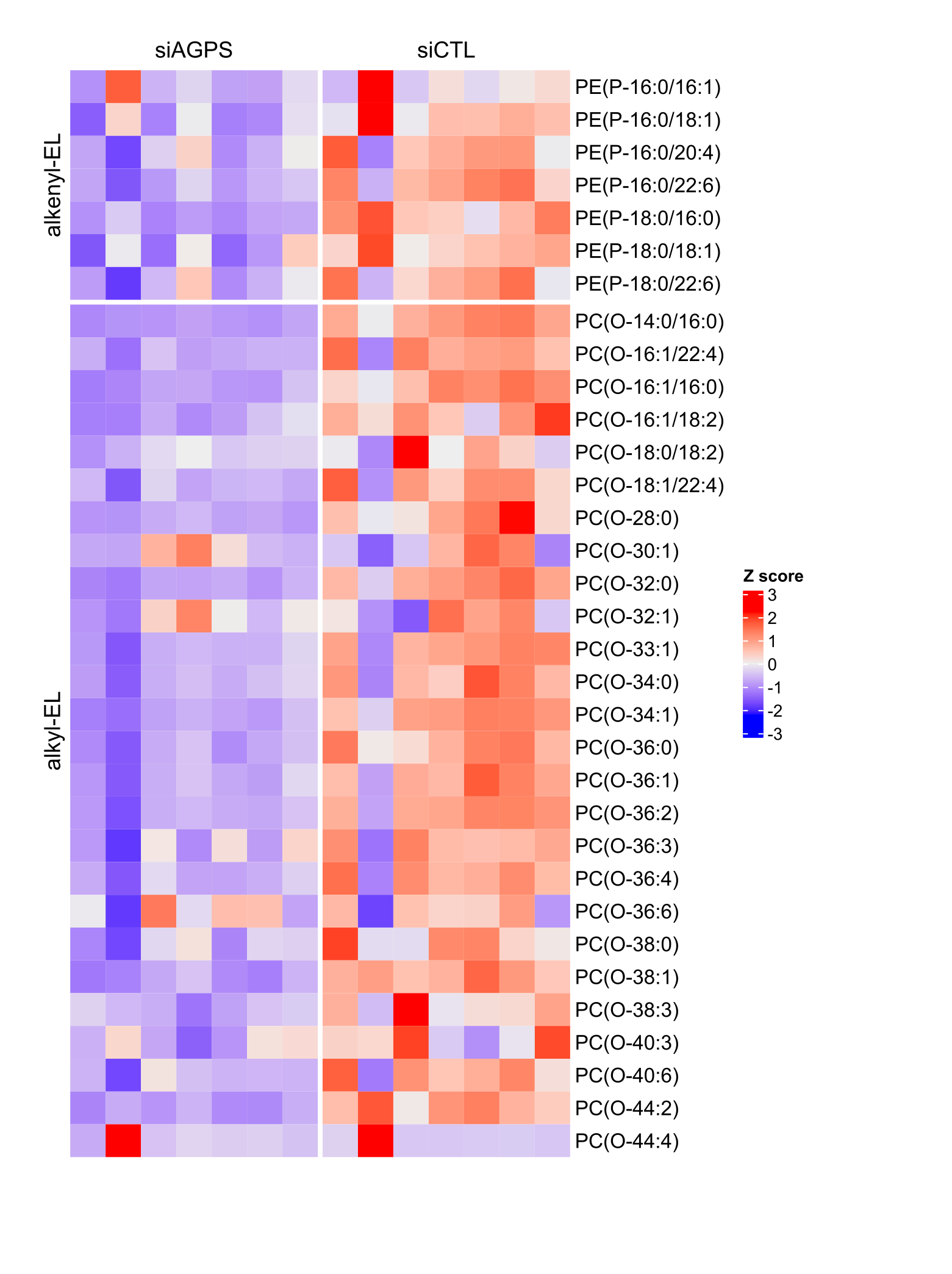

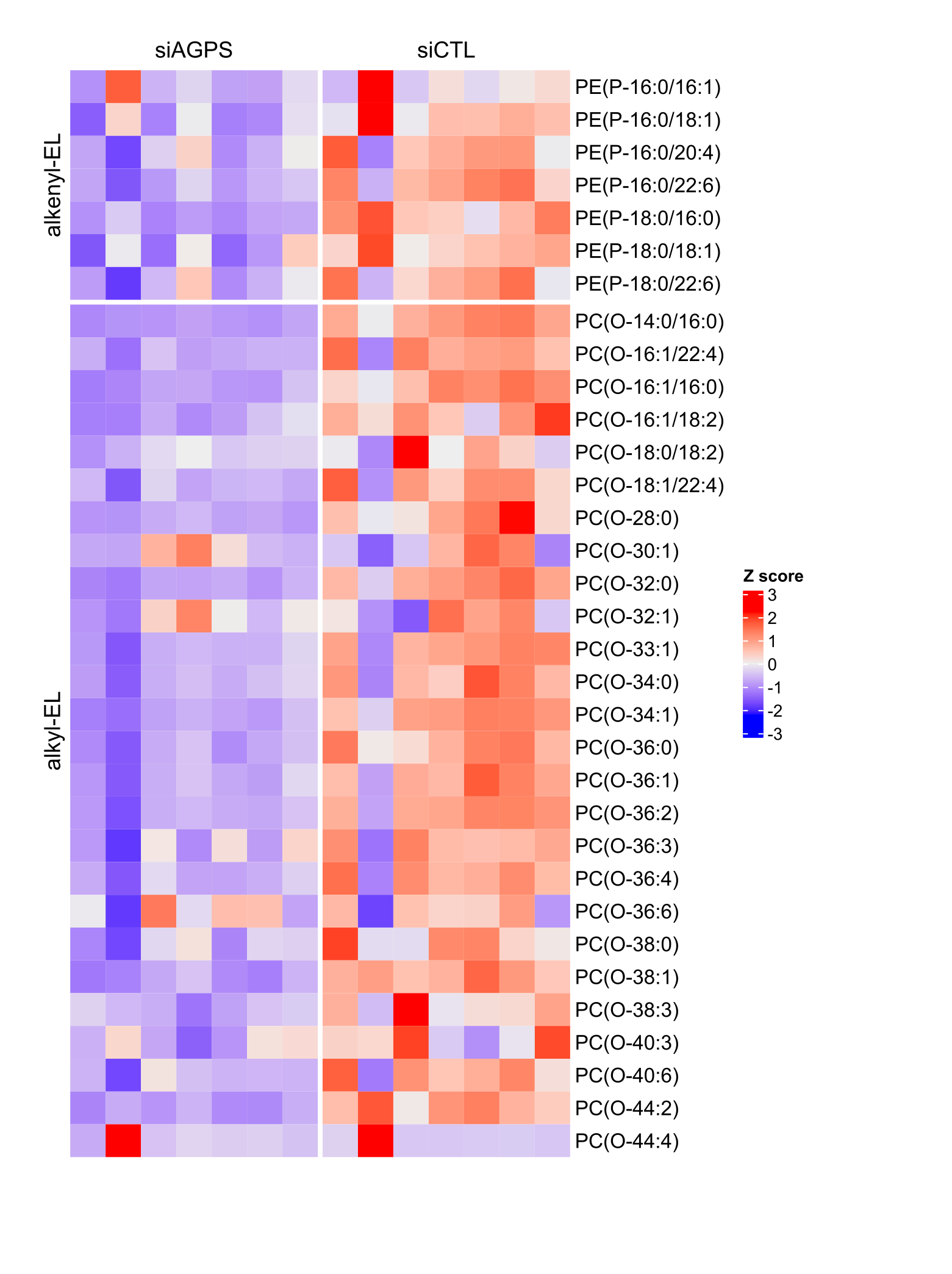


**Supplemental Fig. S12. Heatmap representing the EL species after AGPS knock-down.** MDA-MB-435s cells were tranfected with siCTL or siAGPS#1 during 96h before before lipid extraction (N=7). The heatmap represents the molecular species of EL identified by UHPLC-MS in positive ion mode in the siCTL (left) and siAGPS (right) samples. Each column represents a different sample with siCTL and siAGPS samples working in pairs. Each line represents a different EL (identified on the right) and each colored square represents the Z-score of the EL (see material and methods for details on the Z-scores calculations). High Z-scores (red) indicate a higher quantity of EL in the sample and low Z-scores (blue) a lower quantity.

**Supplemental Fig. S13. The level of alkyl-EL species identified by UHLC-MS (positive mode) are reduced after AGPS knock-down.** MDA-MB-435s cells were transfected with siCTL or siAGPS#1 during 96h before UHPLC-MS experiments in positive ion mode. 7 independent experiments were performed and paired. (N=7; Points, N; bars, median, Wilcoxon signed-rank test, * p<0.05, ns = non significant).

**Supplemental Fig. S14. The level of alkenyl-EL species identified by UHLC-MS (positive mode) are reduced after AGPS knock-down.** MDA-MB-435s cells were transfected with siCTL or siAGPS#1 during 96h before UHPLC-MS experiments in positive ion mode. 7 independent experiments were performed and paired. (N=7; Points, N; bars, median, Wilcoxon signed-rank test, * p<0.05, ns = non significant).

A673

MDA-MB-435s

PC3

**Supplemental Fig. S15. Validation of PEDS1 knockdown by siRNA.**

MDA-MB-435s, PC3 and A673 cells were transfected during 72h or 96h with a SiPED1 or a siCTL and PEDS1 mRNA were analysed by RT-qPCR. Results are relativised to siCTL. Graphics showing median ± interquartile range, Mann-Whitney test, ****p<0.0001, number of independent experiments are indicated in the brackets.

*KCNN3* expression under PEDS1 knockdown

A-673

PC3

C4-2

**Supplemental Fig. S16. *KCNN3* expression is under the control of PEDS1.**

A673, PC3 and C4-2 cells were transfected during 72hwith a SiPED1 or a siCTL and PEDS1 mRNA were analysed by RT-qPCR. Results are relativised to siCTL. Graphics showing median ± interquartile range, Mann-Whitney test, ****p<0.0001, number of independent experiments are indicated in the brackets.

**Supplemental Fig. S17. The levels of EL species identified by UHLC-MS (positive mode) are reduced after PEDS1 knock-down.** MDA-MB-435s cells were transfected with siCTL or siPEDS during 96h before UHPLC-MS experiments in positive ion mode. EL were identified putatively, PC EL were identified only with the sum of their *sn*-1 and *sn*-2 chains. 6 independent experiments were performed and paired. (N=6; Points, N; bars, median, Wilcoxon signed-rank test, * p<0.05, ns = non significant).

**Supplemental Fig. S18. The levels of EL species identified by UHLC-MS (negative mode) are reduced after PEDS1 knock-down.** MDA-MB-435s cells were transfected with siCTL or siPEDS during 96h before UHPLC-MS experiments in negative ion mode. EL were identified putatively, a «_2 » after the EL name represents an isomer. 6 independent experiments were performed and paired. (N=6; Points, N; bars, median, Wilcoxon signed-rank test, * p<0.05, ns = non significant).

**Supplemental Fig. S19. Supplementation with LPC(O-16:0, left) and LPC(P-16:0, right) both increases *KCNN3* expression.** MDA-MB-435s cells were treated daily with 20 µM of EL in liposomes for 96 hours. *KCNN3* mRNA level measured by RT-qPCR is increased after supplementation with LPC(O-16:0, N=4)) or with LPC(P-16:0, N=3) (median ± interquartile range, Mann-Whitney test, **** p<0.0001).

**Supplemental Fig. S20. No effect of acute application of PC(16:0/20:4) on SK3 currents.** Whole-cell currents in HEK293T cells expressing recombinant human SK3 were generated by a ramp protocol from -100 to 100 mV in 500 ms from a constant holding of 0 mV with a pCa 6. Graphs showing the current recorded at 0 mV sensitive to 3µM of PC(16:0/20:4). Data are relativised to the current recorded before lipid application. The line indicates the median, each point represents SK3 current fold change after lipid application to one cell. Points, N ; bars, median. Wilcoxon signed-rank test, ** p<0.001.


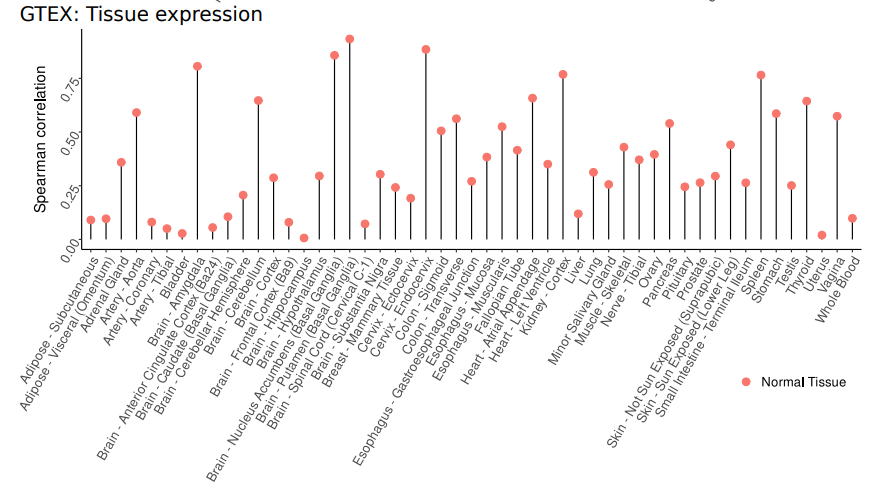


**Supplemental Fig. S21.** Spearman correlation of *KCNN3* and AGPS expression in 51 tissues from the GTEx consortium dataset. R value of the Spearman correlation for the linear regression of KCNN3 and AGPS expression (TPM) were computed for each individual tissue.


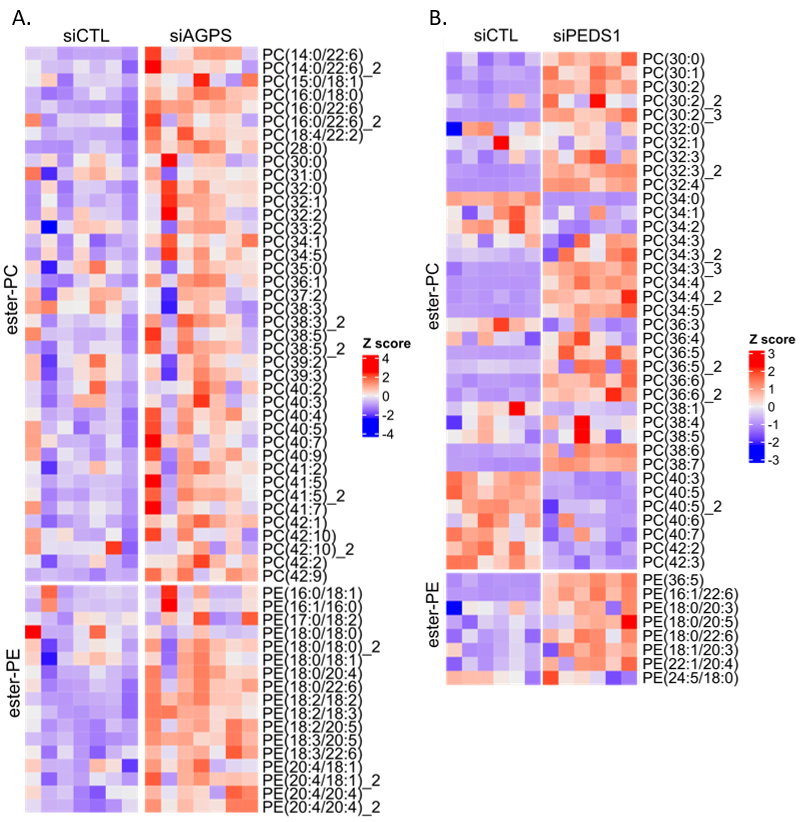


**Supplemental Fig. S22.** **Ester-phospholipid levels following (A) AGPS or (B) PEDS1 knockdown in MDA-MB-435s cells.** Ester-lipids were measured by UHPLC-MS in positive ion mode following AGPS (N=7) or PEDS1 (N=6) knockdown for 96 hours. Each line represents a different lipid (identified on the right) and each colored square represents the Z-score of the lipid (see material and methods for details on the Z-scores calculations). High Z-scores (red) indicate a higher quantity of EL in the sample and low Z-scores (blue) a lower quantity. The notation “_2” or “_3” appended to a lipid name represents isomers.

**EL**


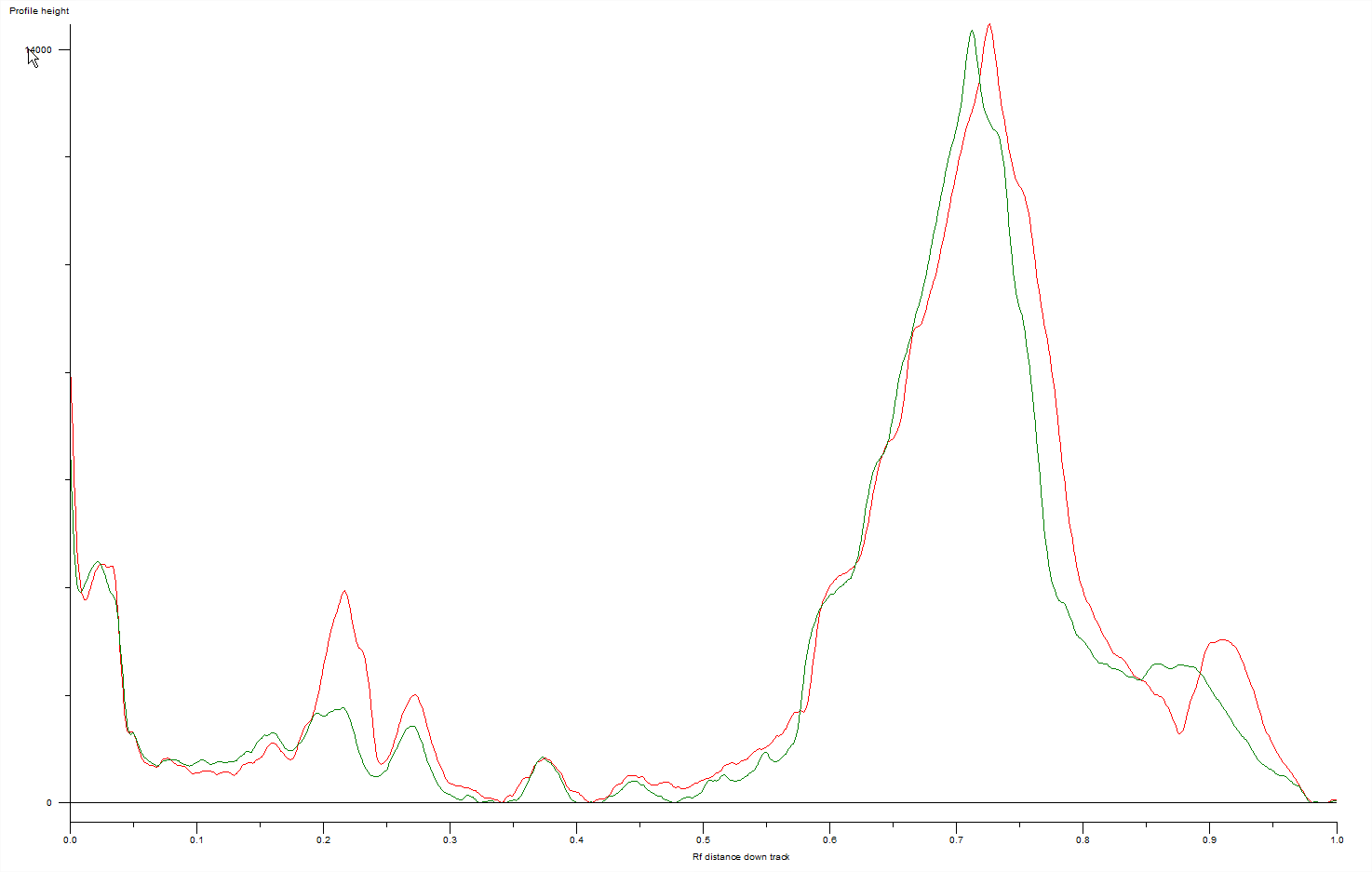

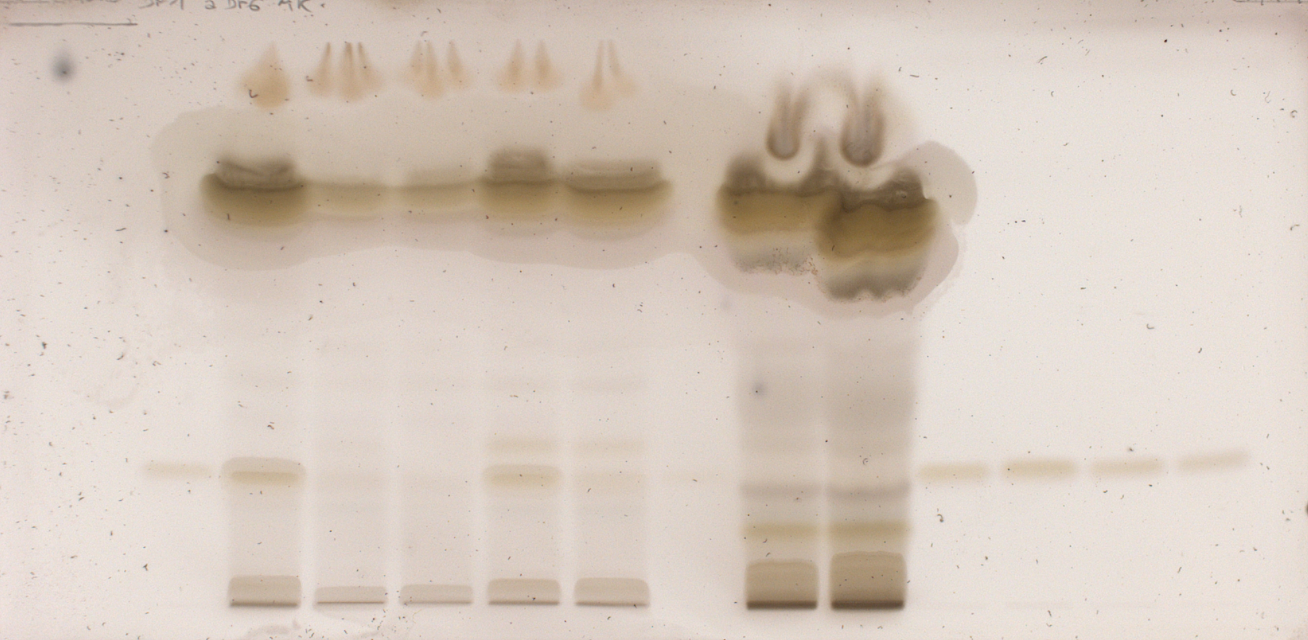


siCTL

siAGPS

siCTL

siAGPS

R_f_

**EL**

1

Lane:

2

3

4

**5**

**6**

7

8

9

10

11

12

A.

B.

**Supplemental Fig. S23. (A).** HPTLC plate for the quantification of EL (alkyl-EL + alkenyl-EL) in siCTL/siAGPS samples. Lanes 5 and 6 correspond respectively to siCTL and siAGPS samples, with the same quantity of reduced lipids deposited in both lanes. Lanes 1, 9-12 correspond to the commercial standard in varying amounts. **(B)** Densitometry from lanes 5 (siCTL, red) and 6 (siAGPS, green).

.
